# Open-Source Selective Laser Sintering (OpenSLS) of Nylon and Biocompatible Polycaprolactone

**DOI:** 10.1371/journal.pone.0147399

**Published:** 2016-02-03

**Authors:** Ian S. Kinstlinger, Andreas Bastian, Samantha J. Paulsen, Daniel H. Hwang, Anderson H. Ta, David R. Yalacki, Tim Schmidt, Jordan S. Miller

**Affiliations:** 1 Department of Bioengineering, Rice University, Houston, Texas, United States of America; 2 Lansing Makers Network, Lansing, Michigan, United States of America; University of Illinois at Chicago, UNITED STATES

## Abstract

Selective Laser Sintering (SLS) is an additive manufacturing process that uses a laser to fuse powdered starting materials into solid 3D structures. Despite the potential for fabrication of complex, high-resolution structures with SLS using diverse starting materials (including biomaterials), prohibitive costs of commercial SLS systems have hindered the wide adoption of this technology in the scientific community. Here, we developed a low-cost, open-source SLS system (OpenSLS) and demonstrated its capacity to fabricate structures in nylon with sub-millimeter features and overhanging regions. Subsequently, we demonstrated fabrication of polycaprolactone (PCL) into macroporous structures such as a diamond lattice. Widespread interest in using PCL for bone tissue engineering suggests that PCL lattices are relevant model scaffold geometries for engineering bone. SLS of materials with large powder grain size (~500 μm) leads to part surfaces with high roughness, so we further introduced a simple vapor-smoothing technique to reduce the surface roughness of sintered PCL structures which further improves their elastic modulus and yield stress. Vapor-smoothed PCL can also be used for sacrificial templating of perfusable fluidic networks within orthogonal materials such as poly(dimethylsiloxane) silicone. Finally, we demonstrated that human mesenchymal stem cells were able to adhere, survive, and differentiate down an osteogenic lineage on sintered and smoothed PCL surfaces, suggesting that OpenSLS has the potential to produce PCL scaffolds useful for cell studies. OpenSLS provides the scientific community with an accessible platform for the study of laser sintering and the fabrication of complex geometries in diverse materials.

## Introduction

The advent of additive manufacturing (AM) technology, also referred to as 3D printing (3DP), has led to its increased use in industry and scientific laboratories, as well as in homes and community makerspaces [1,2]. In AM processes, structures are built up layer-by-layer as a chosen material is patterned precisely via computer instructions into 2D geometries; each patterned layer is built atop the previous layer to yield a 3D structure [3]. This approach to fabrication has been adopted for rapid prototyping of mechanical parts [4,5], manufacturing of small batches of specialized products [6], and fabrication of structures which would be difficult or impossible to otherwise manufacture [7,8]. For example, AM enabled the fabrication of patient-specific medical implants with gradations in internal geometry, which would otherwise be difficult and prohibitively expensive to produce [8]. Two prevalent AM technologies are extrusion 3DP, in which materials are deposited in sequential layers as they are extruded through a small (~0.1–1 mm) aperture [9–14], and stereolithography, which employs a laser or other structured light source to selectively cure a liquid starting material to a solid part [15–19].

Selective Laser Sintering (SLS) is a third and highly versatile AM process that creates solid parts by tracing a laser beam, focused onto a thin layer of powder, in a 2D pattern [20]. The powder is heated and fused into a solid connected pattern as it absorbs the electromagnetic radiation emitted from the laser. 3D structures are fabricated by repeating this process layer-by-layer as new layers of power are laid over the previously fused layers [21]. A key advantage of SLS is the ability to construct overhanging regions which are either inaccessible using other processes or possible only with appropriate support materials. In SLS, unfused powder lying outside of the fused pattern remains within the build volume and acts as support material for subsequent layers, enabling the formation of dramatic overhangs and bifurcations [4]. The use of a focused laser as a patterning tool gives SLS the potential for high (sub-millimeter) feature resolution [4] and the high energy of the laser enables fabrication using not only polymers [22], but also ceramics [23] and metals [24,25]. Recently, SLS has been extended to biomaterials as well [26,27].

SLS has gained attention in the bioengineering community in recent years as a platform for the fabrication of scaffolds for bone tissue engineering, with an emphasis on bioactive and bioresorbable materials [28]. Scaffolds have been fabricated using SLS-based workflows from poly(lactic acid) [29], poly(3-hydroxybutyrate) [30], polycaprolactone (PCL) [31–36], hydroxyapatite [37], and bioactive glasses and ceramics [38,39]. Composite scaffolds have also been fabricated using hydroxyapatite with PCL [40,41], poly(L-lactide) [42], poly(ether-ether-ketone) [43], and high-density poly(ethylene) [44], as well as from β-tricalcium phosphate with PCL [45–47]. Outside of bone tissue engineering, PCL was recently laser sintered into patient-specific airway splints which were implanted in pediatric patients 3–16 months old to prevent airway collapse [48,49]. We sought to utilize SLS for fabrication using biomaterials, but found that SLS machines have remained at industrial scales and price points, due in part to the challenges of controlling process parameters as well as the need for a high energy laser source. Entry-level commercial SLS systems cost upwards of $400,000 and high-end systems range from $800,000 to $1 million.

Here, we describe the development of a low-cost, open-source SLS system (OpenSLS), which makes SLS available to scientists as a highly customizable fabrication platform. We demonstrate the capabilities and limitations of this technology and discuss several possible applications for parts created using OpenSLS. Specifically, we address the production of highly intricate scalable models of physiologic structures, the fabrication of fluidic networks derived from sintered parts, and the implementation of SLS for cell studies aimed at biomaterials science and tissue engineering. We present these diverse applications together to highlight the multifaceted impact that access to low-cost, customizable SLS technology could have on scientific research in various disciplines.

We begin by discussing the design and construction of OpenSLS, which harnesses the features of a commercial laser cutter alongside a simple, custom-built powder distribution module. We first establish fabrication of structures with sub-millimeter features and overhanging regions in nylon, a commonly used material in commercial SLS systems. Subsequently, we demonstrate fabrication of PCL into a diamond lattice structure which could be relevant as a model scaffold geometry for bone tissue engineering. We characterize the accuracy and precision of OpenSLS as well as the surface quality and mechanics of sintered structures and show that the fidelity and quality of sintered parts match those of commercially sintered parts. We also introduce a simple post-processing technique to reduce the surface roughness of sintered PCL structures, with a concomitant increase in elastic modulus and yield stress of these scaffolds. Processed PCL structures are also useful for sacrificial templating of perfusable fluidic networks within orthogonal materials such as poly(dimethylsiloxane) (PDMS) silicone. Finally, we demonstrate that human mesenchymal stem cells seeded onto sintered PCL surfaces were viable and able to undergo osteogenic differentiation and matrix mineralization, suggesting that OpenSLS has the potential to produce PCL scaffolds useful for tissue engineering studies.

We believe that OpenSLS could serve the scientific community as an accessible platform for fabrication of structures composed of a wide range of materials, including those not supported by commercial SLS suppliers or their maintenance contracts. OpenSLS represents a meaningful addition to the growing body of open-source technologies that give engineers and scientists access to and unprecedented control over advanced fabrication and analytical tools at low cost.

## Materials and Methods

### Materials

Selective Laser Sintering was investigated using nylon, a common industrial SLS substrate, and polycaprolactone (PCL). Nylon-12 (PA 650; Advanced Laser Materials, Temple, TX) has a melting point of 181°C, melt-flow index 50 g/10 min, and reported mean particle size 55 μm. PCL (CAPA 6506; Perstorp, Malmö, Sweden; mean molecular weight 50 kDa) has a melting point of 58–60°C, melt-flow index 5.2–11.3 g/10 min, and reported particle size below 600 μm. Materials were used as received from the suppliers.

### Development of OpenSLS

A CO_2_ laser cutter with 60 × 90 cm bed (SeeMeCNC, Goshen, IN) was selected as the base of the OpenSLS system due to its high power range, modular electronics, and large working envelope. A RepRap Arduino Mega Board (RAMBo; Ultimachine, South Pittsburgh, TN), an integrated 3D printer motherboard, was chosen to control OpenSLS due its 5 stepper motor axes, Pulse Width Modulation (PWM) output pins, and compatibility with the widely used open-source Marlin firmware. The laser cutter’s native stepper motors and microstepping drivers were retained for driving its XY gantry, but the motor control lines were re-mapped to motor extension pins on the RAMBo (Fig A in S1 File). While the included 80 W laser tube was compatible with the OpenSLS electronics, the beam did not fire below 2.5 W; thus, it was replaced with a 40 W tube (Automation Technologies, Hoffman Estates, IL) and corresponding 40 W power supply (Lightobject, Sacramento, CA) that provided higher power resolution at lower power settings. Laser control was mapped onto the RAMBo board by locating the enable and control pins on the laser power supply and reconnecting them to PWM pins on the RAMBo board (Fig A in S1 File). We designed the powder handling module (SolidWorks 2013, Dassault Systems, Waltham, MA) and fabricated the majority of its components from laser-cut acrylic and 3D printed parts. Details of the components used in the powder module are provided in the Bill of Materials (S1 Bill of Materials) and on the OpenSLS github repository (github.com/MillerLabFTW/OpenSLS). Caution: laser sintering produces harmful fumes and OpenSLS should only be operated with at least 200 cfm (cubic feet per minute) certified ventilation.

OpenSLS runs on firmware modified from the open-source Marlin Arduino firmware which is widely used in the hobbyist maker community. Marlin (github.com/MarlinFirmware) is designed for extrusion printing, not SLS, so melt extrusion functionalities that are meaningless in an SLS system were removed (e.g. extruder heating, fan control, etc.). Then, G-code commands were assigned for functionalities unique to SLS (e.g. laser firing, powder distribution, etc., see Table A in S1 File). The customized Marlin firmware for OpenSLS may be downloaded from the OpenSLS github repository.

#### 3D model design and fabrication

3D model files were designed and prepared for OpenSLS using a toolchain of open-source software applications developed within the hobbyist maker community. 3D model stereolithography (STL) files were created using OpenSCAD software (openscad.org) or downloaded from an online collection of 3D models (thingiverse.com) The diagrid model and diamond lattice model were listed, respectively, as “Diagrid bracelet” by nervoussystem (n-e-r-v-o-u-s.com), and “Diamond Lattice Model” by pmoews. The reduced diamond lattice was generated by modifying the diamond lattice model in OpenSCAD. Models were sliced into 2D layers as machine-interpretable G-code using Slic3r (slic3r.org; for slicing parameters see Table B in S1 File). The resulting G-code files are incompatible with the motor axis assignments used in OpenSLS, so a custom script was developed to modify the G-code appropriately for SLS (Box A in S1 File). Prints were initiated and monitored through the Printrun Pronterface console (github.com/kliment/Printrun) and laser power settings were set by the user through the Pronterface console. Nylon prints typically used approximately 60 W cm^-2^ (first 1–3 layers) and 30 W cm^-2^ (subsequent layers) while PCL prints used approximately 150 W cm^-2^ (first 1–3 layers) and 100 W cm^-2^ (subsequent layers). Higher power settings for the initial layers helped to ensure adhesion between the print and a layer of painter’s tape which covered the build platform; this adhesion prevented warping throughout fabrication process. The initial powder layer was set by manually creating the thinnest possible uniform coating of powder on the build platform while subsequent layers were created automatically by the powder distributor. Following fabrication, excess powder was removed from sintered parts with compressed air or by tapping the part against the benchtop.

### Analysis of powdered materials and sintered structures

Scanning electron microscopy (SEM) was used to analyze nylon particle size distribution (prior to sintering) as well as for surface analysis of sintered nylon and PCL structures. For particle sizing, sparsely arranged nylon particles were sputter coated with approximately 10 nm gold (Desk V Sputter Coater; Denton Vacuum, Moorsetown, NJ) and imaged on an FEI Quanta 400 Environmental Scanning Electron Microscope (FEI, Houston, TX). PCL particles were too large to be efficiently sized via SEM and were instead sized using an optical stereo microscope (SteREO Discovery.V8; Zeiss, Jena, Germany) after validating that the sizing was consistent with SEM (S2 Fig). For analysis of sintered structures, a representative section was cut away from the structure, sputter coated (~10 nm gold) and imaged. Average surface roughness of sintered PCL (before and after vapor-smoothing) was quantified using SEM images. Images were thresholded in FIJI ImageJ (NIH, Fiji.sc) and MATLAB (MathWorks, Natick, MA) was used to extract the profile of each edge from the thresholded image. The average surface roughness (R_a_) was calculated using the formula $R_{a}=\frac{1}{L}\int_{0}^{L}|Z\left(x\right)|\;dx$, where Z(x) is the edge profile extracted in MATLAB and L is the length of the edge.

Microcomputed tomography (μCT) was used to analyze the internal structure of sintered parts. Samples were scanned in 0.5 degree steps at 20 μm resolution on a SkyScan 1272 X-ray microCT scanner (Bruker USA, Billerica, MA) equipped with 11 megapixel detector. The X-ray source was set to 50 kV and 200 μA with an exposure duration of 424 ms. Image reconstruction was performed in nRecon software (Bruker) and volumetric visualization was performed in CTVox software (Bruker). μCT data is presented as virtual cross-sections through the volumetric scan, performed in CTVox software.

### Vapor-smoothing of PCL with dichloromethane

Sintered PCL structures were suspended from the rubber stopper of a 4 L filter flask. Dichloromethane (DCM; Sigma-Aldrich, St. Louis, MO) was added to a round-bottom flask with stopcock and the stopcock was connected to the vacuum adapter on the filter flask. DCM was heated until boiling, then the stopcock was opened to allow DCM vapor to diffuse into the filter flask. The suspended PCL structure was monitored and removed after it appeared sufficiently smoothed (3–5 minutes, typically). Overexposure to DCM vapor led to deformation of PCL structures due to gravity. Segregation of the liquid DCM in a separate chamber from the specimen was critical for achieving uniform smoothing of exterior and interior surfaces.

### Dimensional accuracy and precision

Two geometries were used to assess dimensional accuracy and precision of nylon and PCL structures. The first was a 10×10 mm cube with 1×1 mm rectangular studs on each face. The 10mm and 1mm features were measured with calipers for 10 samples produced over 3 sintering runs. The second geometry was the full (nylon) or reduced (PCL) diamond lattice model. Lattices were μCT scanned as described above, reconstructed, and exported as STL files. 3D scans were aligned with their corresponding models using Blender (blender.org) and Geomagic Control (3D Systems, Rock Hill, SC). Aligned scans and models were divided into corresponding 100μm slices using Creation Workshop (envisionlabs.net). A custom MATLAB script was used to quantify overlapping and non-overlapping pixels in each slice for each scan/model pair.

### Mechanical testing

Uniaxial compression testing was conducted in accordance with a modified version of ASTM standard D695-02a. Test samples were cylinders 12.7 mm in diameter and 25.4 mm in height with a 3D rectilinear macroporous network geometry as described by Eshraghi and Das [36]. Samples were subjected to compressive loading on a mechanical testing system (MTS, 858 Mini Bionix, Eden Prairie, MN) equipped with a 10 kN load cell. Samples were compressed along their long axes by two parallel plates at a cross-head rate of 0.5 mm/min after a preload of 25 N was applied. Applied load and displacement were measured and later converted to stress and strain based on the sample dimensions. Elastic modulus was measured as the slope of the linear region of the stress-strain curve, yield stress was measured as the maximum stress before failure, and strain at yield was measured as the corresponding strain.

### Sacrificial templating of sintered PCL structures

Sintered PCL structures were vapor-smoothed in DCM and embedded in poly(dimethyl siloxane) (PDMS; Sylgard 184, Dow Corning, Midland, MI). PDMS (10:1 PDMS base to curing agent) was allowed to cure for 48 hours in a 3D-printed container made from poly(lactic acid) (PLA). After curing, the container and PDMS-embedded PCL were stirred overnight in DCM to dissolve the PLA and PCL. The resulting structure was rinsed with 99% isopropyl alcohol (IPA), then soaked in an IPA bath. The internal void space generated by the sintered PCL was visualized by perfusion with a solution of food coloring dye in IPA and by μCT scan.

### Morphology of human mesenchymal stem cells on sintered PCL

Human Mesenchymal Stem Cells (hMSC; kindly provided by Rooster Bio, Frederick, MD) were stably transduced at passage 1 with a second-generation lentivirus in accordance with Rice University Institutional Biosafety Committee oversight on Protocol #662023. Methods for lentiviral transduction were modified from Kutner and colleagues [50]. Lentiviral transduction resulted in dually labeled cells expressing green fluorescent protein (eGFP) in the cytoplasm and H2B-mCherry in the nucleus. Labeled cells were considered preferable to staining (e.g. with DAPI/Phalloidin) because constitutive labeling allows live imaging at unlimited time points, avoids limitations due to diffusion of dye molecules, and avoids additional stress to cells caused by rinse steps associated with staining. hMSCs were maintained at 37°C, 5% CO_2_ in DMEM (Corning, Manassas, VA) supplemented with 10% fetal bovine serum (Atlanta Biologicals, Flowery Branch, GA) and 1% antibiotic (Penicillin/Streptomycin; Gibco Life Technologies, Grand Island, NY). PCL platforms (approximately 8×5×2 mm) were sintered and sterilized in 70% ethanol. Half of the PCL platforms were vapor-smoothed in DCM prior to sterilizing and seeding. Labeled hMSCs (passage 5) were seeded on the platforms at a density of 3×10^6^ cells cm^-2^ in an 80 μL droplet of media. For imaging on an inverted microscope, the platforms were inverted in 24-well plate wells. hMSCs on the platforms were imaged at 3, 7, and 10 days on a Nikon Eclipse Ti inverted epifluorescent microscope (Nikon USA) equipped with a Zyla 4.2 sCMOS camera (Andor, South Windsor, CT). Images were acquired in z-stacks to compensate for the uneven surface of the PCL, then focus-stacked using open-source Enfuse software (enblend.sourceforge.net). ImageJ was used to remove background autofluorescence from the PCL and to perform gamma correction to improve cell visualization.

### Viability of hMSCs on sintered PCL

Unlabeled hMSCs (passage 4–6) were seeded on vapor-smoothed PCL platforms at a density of 3×10^5^ cells cm^-2^ as described above. After 1 day in culture, the platforms were rinsed in PBS and live/dead staining was performed in accordance with the manufacturer’s instructions (Kit #R37601, Thermo Fisher Scientific, Waltham, MA). PCL boats treated with 70% ethanol prior to staining were used as negative controls. Fluorescent imaging was performed as described above, using exposure times determined by imaging live and ethanol-treated hMSCs on tissue culture plastic. Live and dead cells were counted either manually or using ImageJ and results encompassed three PCL platforms seeded in separate experiments.

### Osteogenic differentiation of hMSCs on sintered PCL

Unlabeled hMSCs (passage 2) were seeded on sintered PCL platforms (unsmoothed and vapor-smoothed) as described above. After 1 week incubation in growth media, half of the platforms were changed to osteogenic media, consisting of growth media supplemented with 10 mM β-glycerophosphate and 50 μg/mL L-ascorbic acid (Sigma). The remaining platforms continues to be incubated in normal growth media. 32 days after the switch to osteogenic media, Alizarin Red S staining was performed using a modified protocol from Madurantakam and colleagues [51]. Briefly, PCL platforms were fixed in 4% paraformaldehyde, rinsed thrice with PBS, and incubated in 2% Alizarin Red S solution (Sigma) for 30 minutes. Stained platforms were repeatedly rinsed in PBS until no further dye could be removed, and then photographed. Dye was solubilized by incubating the PCL platforms in 50% acetic acid overnight, then diluted 10-fold in PBS. Dye concentration was obtained by absorbance scan at 424 nm following an absorbance sweep to determine the wavelength of maximum absorbance (Tecan microplate reader; Tecan, Mannedorf, Switzerland).

## Results and Discussion

### Development of OpenSLS

**Fig 1:**
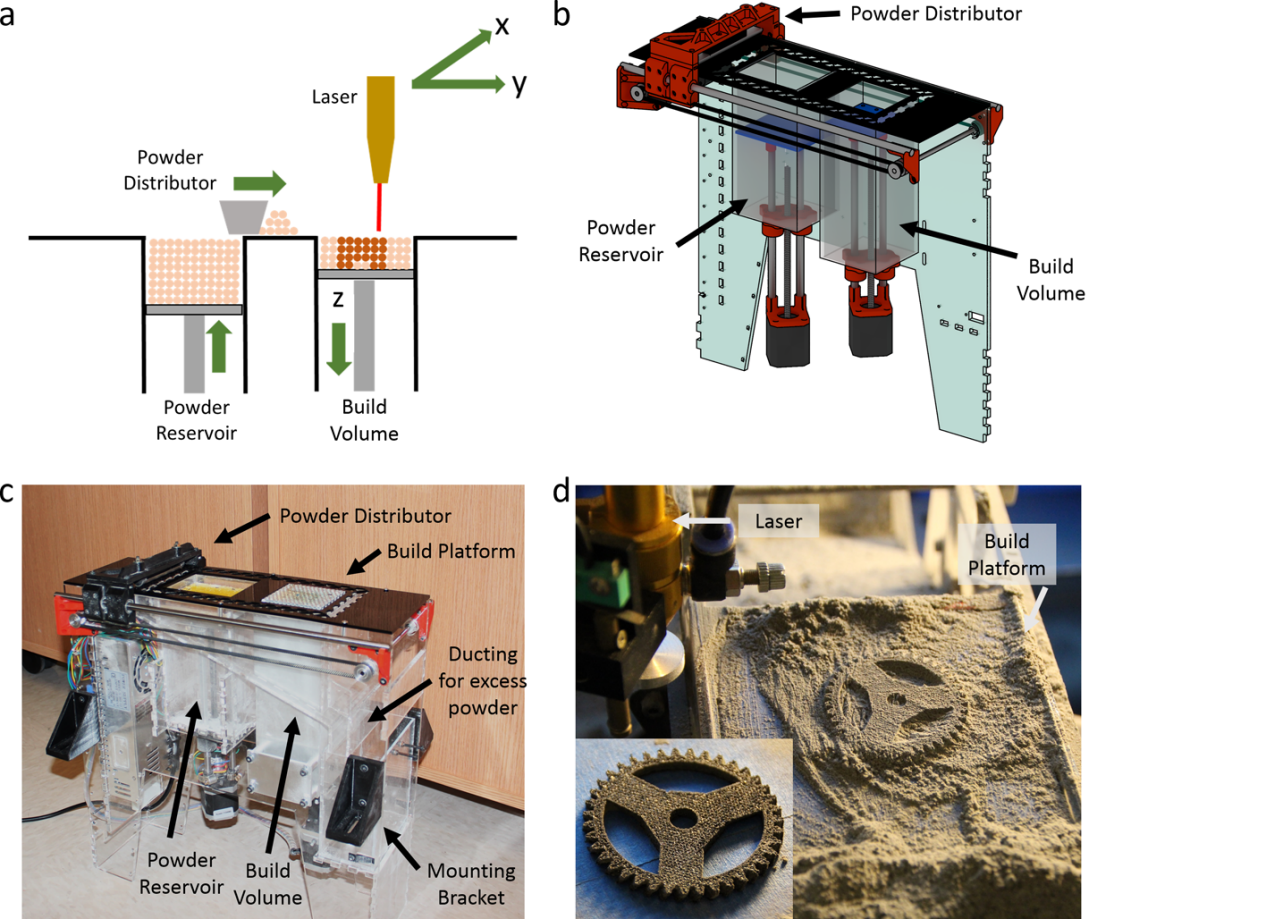
Custom Open-source Selective Laser Sintering (OpenSLS) hardware. a) A simplified depiction of the SLS process illustrates the sintering of powdered materials into 3D parts using a laser. For each new layer, the powder reservoir piston moves up to expose a layer of fresh powder while the build platform lowers within the build volume to leave space for the new powder layer at the top. The distributor pushes the exposed powder from the reservoir to the top of the build area so that the laser can pattern the next layer. b) A schematic rendering of our custom powder handling module. All of the red parts are 3D printed; full designs for these and the laser-cut acrylic walls may be found on the OpenSLS github repository. With the exception of the blue-green wall in the background, the exterior acrylic walls (as well as the exit ducts for excess powder) have been omitted for clarity. c) A photograph of the assembled powder module that was used throughout this study shows the components highlighted in the schematic (b) as well as the remaining acrylic walls and ducting for excess powder. The powder module was readily integrated into a commercial laser cutter with the indicated mounting brackets. d) After mounting the powder module in the laser cutter, we successfully implemented selective laser sintering and fabricated structures such as the illustrated gear. The gear is shown just after sintering and powder removal as well as after cleaning with compressed air (inset).

The lack of affordable laser sintering technology for a laboratory setting motivated the construction of an inexpensive open-source Selective Laser Sintering (OpenSLS) system repurposed from a commercial laser cutter. The native functionality of the laser cutter, in combination with a custom-designed powder handling module, creates the fundamental hardware necessary for SLS (schematized in Fig 1A). SLS requires a laser to pattern desired geometries in powdered materials, a build platform where this patterning occurs, a reservoir to hold the powdered substrate, and a distributor to carry powder from the reservoir to the build platform. Laser cutters are widely available and affordable laser patterning tools common in laboratories, machine shops, and makerspaces. CO_2_ laser cutters use the same wavelength laser (10.6 μm) as commercial SLS systems and are capable of positioning a fine laser spot (approximately 375 μm in our system) with similar spatial precision. While conventional systems use optics to raster scan a laser beam across the powder bed, the laser in OpenSLS travels along a toolpath corresponding to the geometry being sintered.

We implemented the powder handling requirements for SLS with a simple powder-handling module that drops into a laser cutter. Together, the laser cutter and powder-handling module create a customizable platform for SLS research. To unify control over the laser positioning system and the powder-handling module, we overrode the built-in electronics of the laser cutter and controlled all electronics with an open-source RAMBo motherboard (Fig A in S1 File). It should be noted that OpenSLS does not currently embody the full set of features found in commercial SLS systems, namely pre-heating of powders and inert gas sintering environment.

The powder-handling module (Fig 1B and 1C) consists largely of laser-cut parts, allowing it to be fabricated using the same laser cutter that will serve as the laser patterning tool in OpenSLS. 3D-printed parts (colored red in Fig 1B) are also used extensively and all other hardware is readily available. The cost to build OpenSLS was approximately $2,000 plus the cost of the laser cutter. A github repository contains design files for the laser-cut and 3D-printed parts which we designed for OpenSLS (github.com/MillerLabFTW/OpenSLS), and details of the remaining components used are provided in the Bill of Materials (S1 Bill of Materials). The powder handling module is structured around two rectangular pistons: a build platform and a powder reservoir. These pistons translate vertically, allowing the powder reservoir to expose powder for distribution and the build platform to lower within the build volume to accommodate the next powder layer. Powder is moved from the reservoir to the build platform by a curved metal spatula mounted on a 3D-printed distributor. Excess powder moved by the distributor travels through acrylic ducting and is re-collected in a hopper (visible in Fig 1C) for recycling.

The electronics and firmware which control OpenSLS were developed using an existing open-source electronics motherboard (RAMBo) with open-source firmware. A wiring diagram for OpenSLS is provided (Fig A in S1 File), as is the custom firmware that we developed (OpenSLS github repository). After developing the OpenSLS powder module, integrating it with a laser cutter, and implementing control via open-source electronics and firmware, we were able to fabricate simple geometries using thermoplastic powders (Fig 1D). When sintering polymeric powders, we observed temporary formation of a liquid melt pool in the region of powder exposed to the laser, indicating that OpenSLS bonds powder particles via a full melting mechanism (S1 Video) [52].

### Selective Laser Sintering of nylon

**Fig 2:**
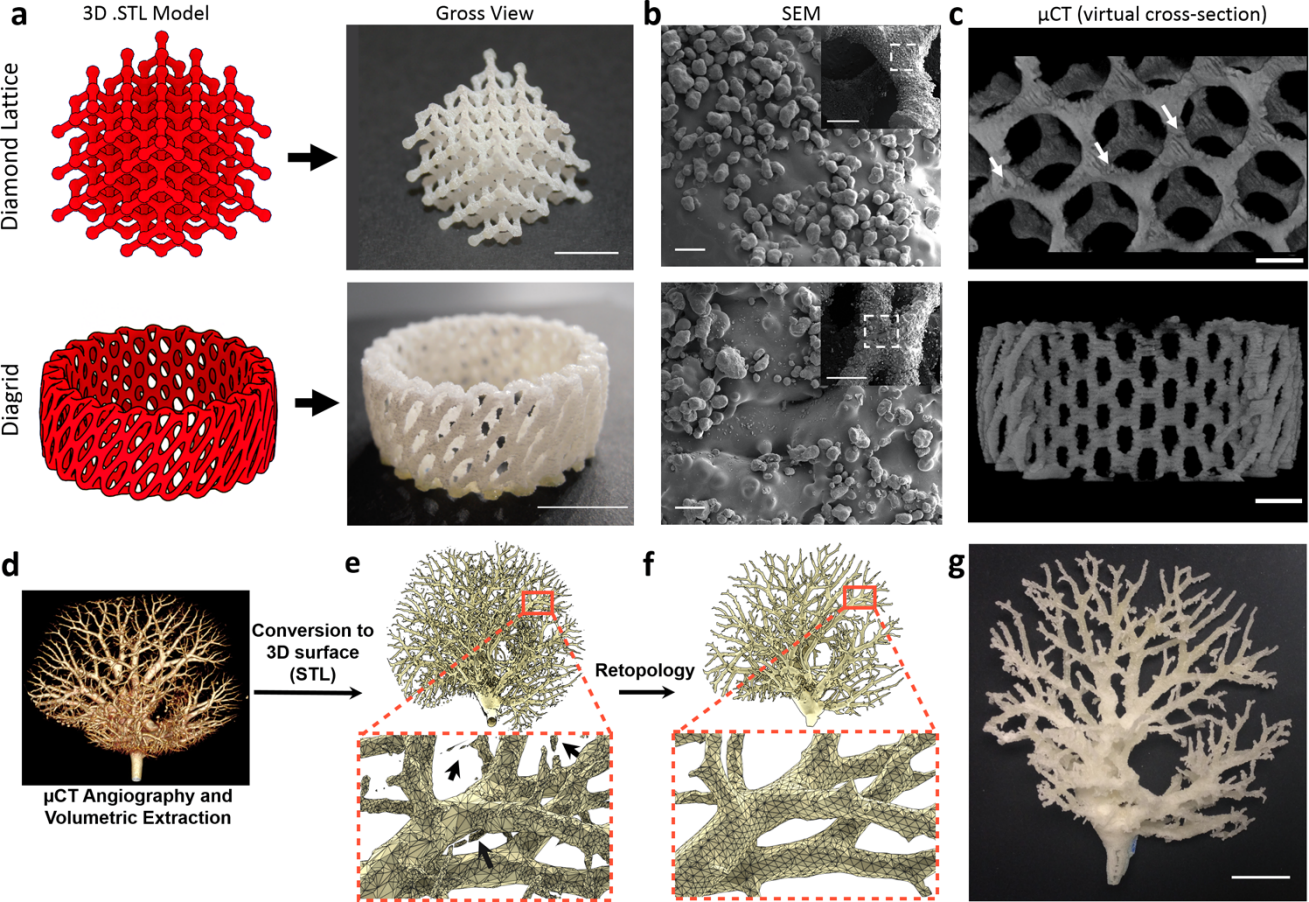
Complex geometries fabricated in nylon with OpenSLS. a) Two representative models were sintered in nylon (mean particle size = 46 ± 20 μm, see S1 Fig) with 150 μm layer height, resulting in reproduction of the features and dimensions of the original geometry (scale bars = 1 cm). b) SEM imaging showed a smooth surface, partially covered by unfused nylon particles (160x magnification, scale bars = 100 μm). Inset for b) Lower magnification SEM images of structures with white box around the magnified region (scale bars = 1 mm). c) Microcomputed tomography (μCT) scans reveal that the interiors of sintered nylon filaments have small, irregular cavities (white arrows) dispersed within a predominantly fused core (scale bars = 3 mm). d-g) Here we demonstrate the ability to fabricate complex structures extracted from biological data. The architecture of the arterial vascular tree was extracted from a μCT scan of a mouse liver (d,e) and this raw data was retopologized to make the model sinterable (f). Black arrows in (e) indicate regions of disconnected (non-manifold) geometry that were removed through the retopology process. 2D mouse liver scans were courtesy of Chris Chen and Sangeeta Bhatia, additional research available via [53]. The liver vasculature was scaled up in size and sintered in nylon (g), illustrating the capacity of OpenSLS to fabricate geometries with extreme overhanging regions (scale bar = 1 cm).

We laser sintered powdered nylon to validate the ability of OpenSLS to consistently produce solid parts with high resolution and to evaluate the system’s performance (Fig 2). A diamond lattice model and a diagrid model were chosen as representative geometries for sintering and we successfully fabricated them in nylon (Fig 2A). Inexpensive desktop 3D printers could fabricate these geometries at their original scale; however, we scaled the diamond lattice and diagrid to 20% and 30% of their initial dimensions, respectively, to highlight the capacity of OpenSLS to fabricate features at the sub-millimeter scale. A video recorded while sintering the diagrid model in nylon illustrates the fusion of powdered materials into a solid part, as well as the distribution of a new layer of powder (S1 Video).

The parameter space within OpenSLS includes the material, powder layer height, laser speed, and laser power. Optimizing the last two parameters was the primary challenge in achieving consistent sintering with high feature resolution. When the power is too high, over-sintering may occur such that material lying outside of the laser toolpath is fused along with the intended pattern. Additionally, excessive fusion between powder layers may lower the resolution along the build axis of the part. Insufficient laser scanning speed may result in an irregular melt pool as the powder is sintered, leading to distortions or cavities which reduce print quality [54]. Alternatively, when the power is too low or the laser speed is too fast, the material may not form a continuously fused feature. These circumstances are one cause of the balling defect in SLS: when insufficiently melted, some powdered materials tend to ball up into disconnected spheres instead of contiguous filaments [55,56]. While print time may be lowered by printing with fast laser speed and compensating with high power, we found that higher print quality was obtained by reducing laser speed (10–30 mm/s) at lower power (30–60 W cm^-2^ in nylon). Importantly, the power and speed settings, as well as the layer height, need to be independently optimized for different materials.

From gross examination, nylon appeared to sinter with smooth exterior surfaces. Under SEM examination, we observed a smooth core of fused nylon, with a partial layer of unfused particles loosely attached (Fig 2B). This surface morphology is reasonably consistent with nylon parts sintered on commercial SLS systems [57]. The thickness of the partial outside layer is dictated by the particle size of the powdered material–about 60μm for nylon (S1 Fig). To study the interior of the fused core regions, we captured microcomputed tomography (μCT) scans of the diamond lattice and diagrid. Virtual cross-sections through the scans provide additional evidence that the sintered nylon struts consist primarily of a solid fused core (Fig 2C). We did note that the uniformly fused core was disrupted occasionally by small, irregular cavities. Such defects in the otherwise smooth core are likely a consequence of steep thermal gradients induced by rapid, localized heating from the laser, which are known to introduce stress and deformation in fabricated structures [58]. For this reason, commercial systems typically pre-heat the powder just below its melting point so that minimal energy must be added from the laser and thermal stress is reduced during sintering [59]. Powder pre-heating has not yet been implemented in OpenSLS, but the construction of the build volume using aluminum enables the introduction of a heating element without major alterations to the design.

Amongst bioengineers and medical professionals, there is interest in reproducing physiological structures captured via medical imaging techniques [60–62]. For example, surgeons increasingly use 3D-printed surgical guides for pre-operative planning since they provide a 3D representation of the patient-specific anatomy that the physician will manipulate [63–67]. We sought to illustrate a scheme for reproducing physiologic structures obtained through medical imaging and identified the architecture of liver vasculature as a model geometry. Liver vasculature contains dramatic overhanging regions as well as highly branching features that are well suited for SLS fabrication. The raw biological data, extracted from μCT angiography of a mouse liver [53], contained artifacts including uneven surfaces and geometry detached from the primary contiguous structure (Fig 2E). Computational retopology of the model resulted in the removal of disconnected features and irregular surfaces to create a manifold geometry for SLS (Fig 2F). Some structural detail is lost during the retopology process such that the original biological structure is approximately but not precisely preserved in the retopologized model. Successful laser sintering of the retopologized vasculature in nylon demonstrates the potential for OpenSLS to fabricate branching, overhanging structures originating from medical imaging data (Fig 2G).

### SLS and vapor-smoothing of polycaprolactone

**Fig 3:**
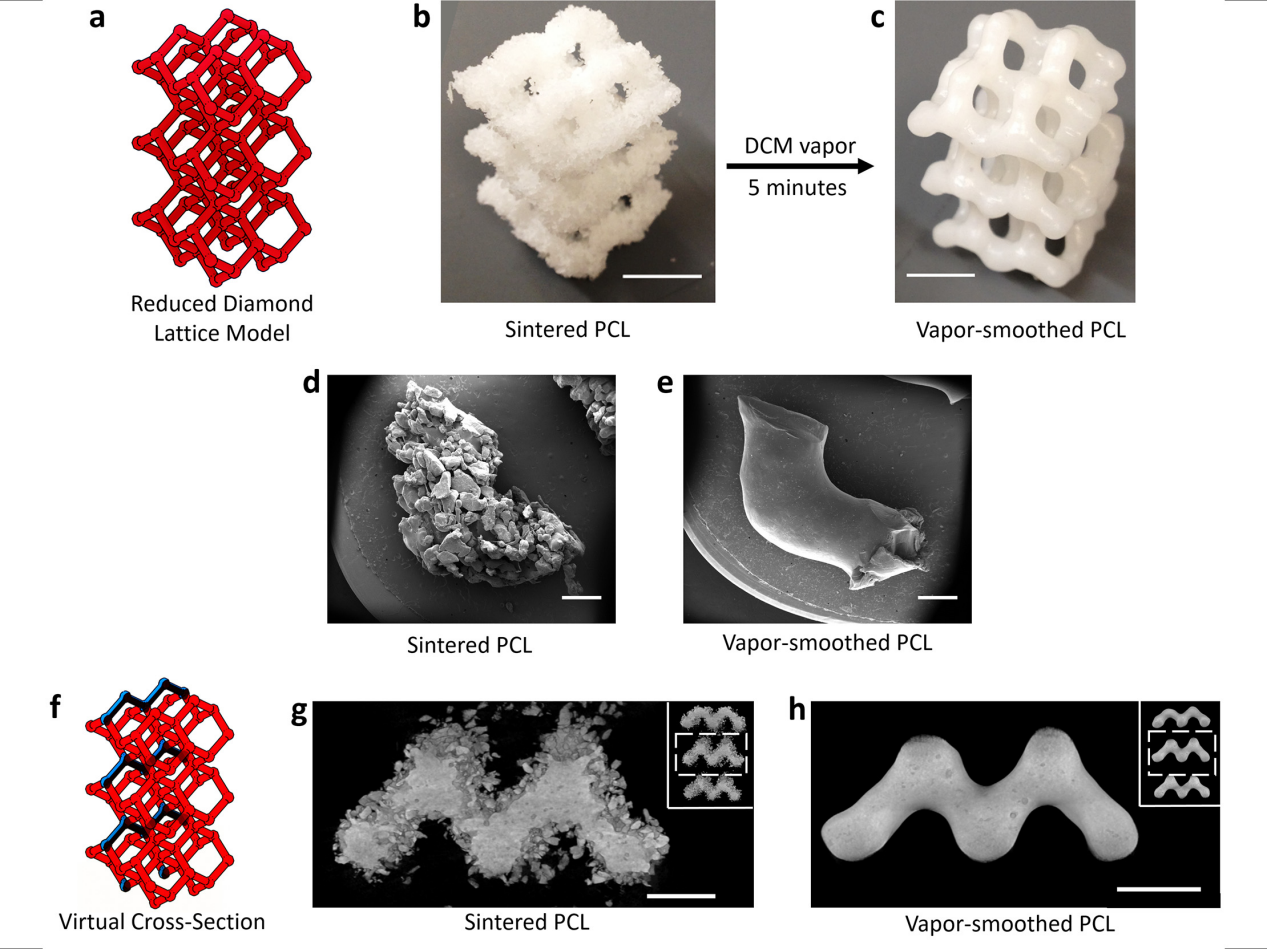
Surface and volumetric analysis of sintered and vapor-smoothed polycaprolactone (PCL) structures. a-c) A reduced diamond lattice model (a) was sintered in PCL (average particle size = 517 ± 172 μm, see S1 Fig) with 300μm layer height. Sintered PCL lattices (b, scale bar = 1 cm) were exposed to a vapor bath of DCM resulting in a smooth surface finish (c; scale bar = 1 cm). d,e) SEM images of struts cut away from unsmoothed (d) and vapor-smoothed (e) lattices demonstrate that while sintered PCL exhibits a rough surface composed of discrete, irregular PCL particles, vapor smoothing results in a smooth, uniform surface devoid of any unfused PCL (scale bars = 1 mm). f-h) A virtual cross-section through μCT scans (schematized in (f)) shows that the surface of sintered PCL (g) is dominated by loosely attached, unfused particles surrounding a fused core containing some irregular cavities. The scan after vapor smoothing (h) confirms that the fused core is undisturbed by the vapor smoothing process (scale bars = 5 mm). Inset for g,h: full μCT virtual cross-section, white box indicates the magnified region.

Following the many studies that used commercial SLS systems to fabricate macroporous polycaprolactone (PCL) structures [31–36,40,45], we explored the possibility of sintering a diamond lattice geometry in PCL. We purchased PCL with the finest grain size available and measured the average size of a PCL particle to be 517 ± 172 μm (S1 Fig). In SLS, the resolution of sintered parts is limited by either the spot size of the laser (375 μm in our system) or the size of the starting powder grains, whichever is larger. Because PCL has a much larger particle size than typical powders used in SLS, it exhibits considerably lower resolution when sintered. Indeed, a diamond lattice model sintered in PCL displayed a high degree of surface roughness upon gross examination (Fig 3B). Despite attempts to cryogenically mill and grind the PCL powder, we were unable to noticeably reduce its grain size to improve the resolution of PCL surfaces. Previously, a PCL blend with average particle size <150 μm was available (CAPA 6501, Perstorp), however, this product has been discontinued. Larger PCL grains, such as the CAPA 6506 used in this study, require advanced jet milling to be effectively reduced to this size [49].

In lieu of powder size reduction, we developed a surface smoothing technique which uses dichloromethane (DCM) vapor to reduce the surface roughness of sintered PCL (Fig 3C). The DCM vapor solvates PCL crystals protruding from the sintered structure; the surface is smoothed as the solvanted PCL minimizes its exposed surface area under surface tension. When exposed to DCM vapor, the surfaces of sintered PCL typically became smooth and glossy in 3–5 minutes (S2 Video). This approach is similar to one used by 3DP enthusiasts who smooth parts made of acrylonitrile butadiene styrene (ABS) using acetone vapor or parts made of poly(lactic acid) (PLA) using DCM [68]. Analysis of PCL lattices via SEM revealed that the surface of sintered PCL is composed of discrete particles with irregular shape consistent with the powdered PCL (Fig 3D). In contrast, vapor-smoothing imparts a uniformly smooth surface devoid of any unfused particles (Fig 3E). Scans of PCL diamond lattices using μCT indicated that the vapor-smoothing process does not disturb the interiors of sintered filaments, which are completely fused except for small sporadic cavities (Fig 3G and 3H).

### Characterization of OpenSLS process

OpenSLS was evaluated as a manufacturing process through dimensional characterization of sintered parts as well as by mechanical testing. As discussed above, the laser spot size determines the resolution (and therefore the dimensional accuracy) of sintered parts when small particles such as nylon (~50 μm) are used. Therefore, the dimensional accuracy and precision of nylon (Table 1) approach the maximum accuracy and precision for the entire OpenSLS system. Nylon dimensions were accurate within <100 μm in the X and Y directions for centimeter-scale nominal dimensions, reflecting the intrinsic accuracy of the XY positioning system of the laser cutter. These results are consistent with the accuracy and precision found for nylon sintering on commercial SLS systems (250 μm) [69,70]. Z-dimensions were somewhat less accurate (<500 μm), but this could be simply improved by rescaling the nominal part dimensions as part of the 3D model preparation workflow. Unsurprisingly, accuracy was slightly poorer for millimeter-scale nominal dimensions; however, the precision (indicated by the standard deviation) was comparable. Overall, the accuracy and reproducibility of OpenSLS are sufficient to produce nylon parts at or below the millimeter scale with sub-millimeter tolerance.

**Table 1:** Dimensional Accuracy and Precision of OpenSLS. Note: Measurements are reported as mean ± SD

	10 mm nominal dimension		1 mm nominal dimension	
	Measured x-y dimension (mm)	Measured z dimension (mm)	Measured x-y dimension (mm)	Measured z dimension (mm)
**Nylon**	10.03 ± 0.06	10.31 ± 0.15	1.14 ± 0.08	1.07 ± 0.09
**Sintered PCL**	11.95 ± 0.22	11.08 ± 0.25	2.33 ± 0.23	2.09 ± 0.20
**Smoothed PCL**	11.81 ± 0.26	10.86 ± 0.28	2.29 ± 0.22	2.10 ± 0.18

The measured dimensions of PCL are less reflective of the accuracy and precision of OpenSLS because the large particle size (~500 μm) limits the resolution. PCL measured dimensions consistently exceeded their nominal length by 1–2 mm for both centimeter- and millimeter-scale features, which is consistent with our observation that large PCL particles become partially fused at the edges of sintered structures and protrude beyond the nominal dimensions. Unsurprisingly, these results demonstrate poor accuracy compared to previous studies which used more finely milled PCL [35] and highlight the contribution of the raw material size to the dimensional accuracy of the resulting sintered part. Vapor-smoothing slightly improves the accuracy of PCL sintering since protruding grains of PCL are solvated and pulled towards the surface. However, this effect was not statistically significant and the smoothed parts exhibited, on average, lower precision than their unsmoothed counterparts.

**Fig 4:**
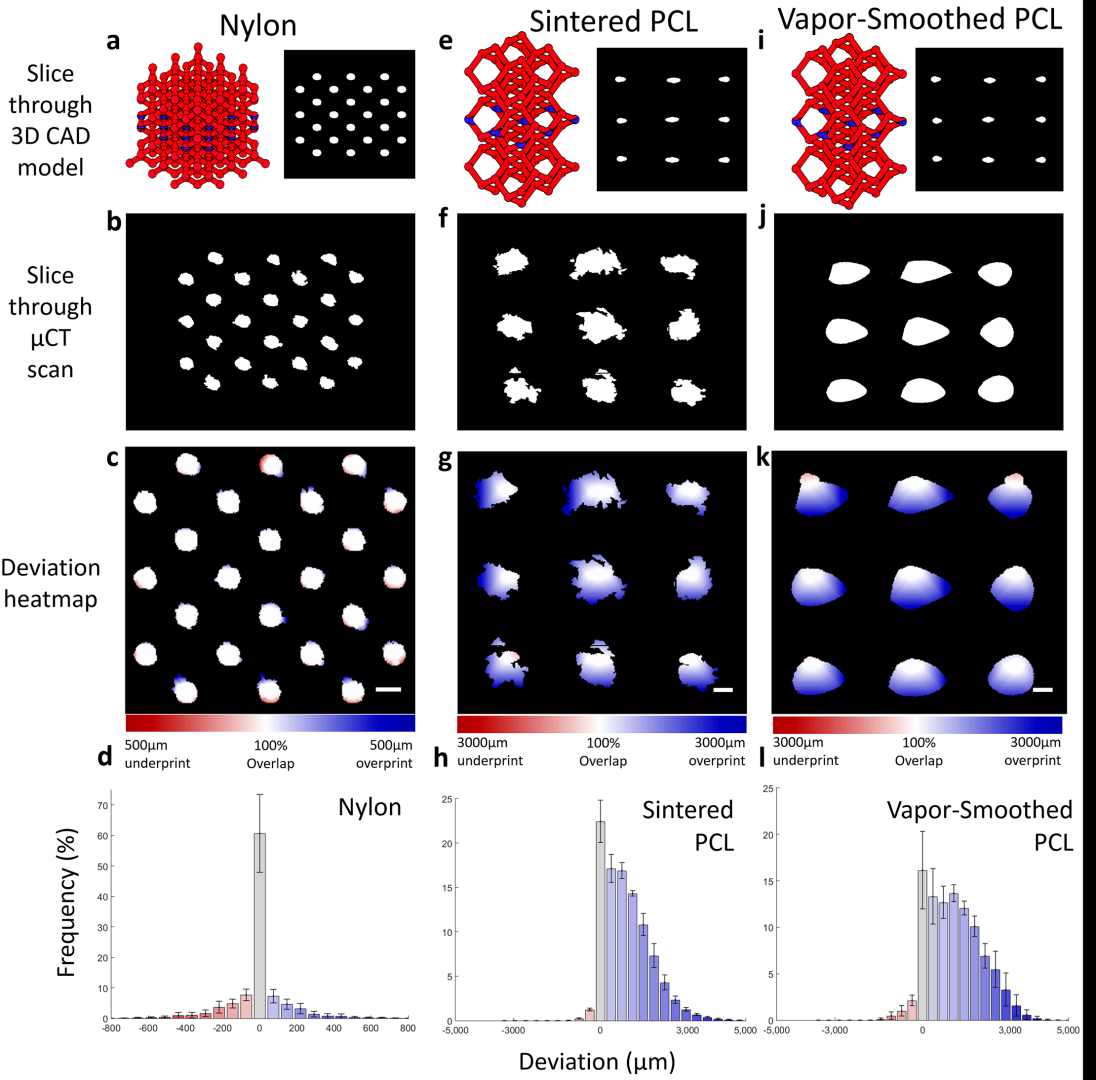
Dimensional fidelity of sintered nylon and PCL. μCT scans of sintered diamond lattices were compared slice-by-slice to their corresponding CAD models to quantify the fidelity of OpenSLS. a,e,i) Left: CAD rendering of full (a) and reduced (e,i) diamond lattices with a representative slice indicated in blue. Right: Cross-section view of the selected slice. b,f,j) Corresponding slices through μCT scans of sintered diamond lattices indicate that nylon (b) falls closely within the area of its original model, while both unsmoothed (f) and smoothed (j) PCL substantially exceed the print area of their original models. c,g,k) Heatmaps of the deviation between scans and models show that for nylon, there are regions of both under- and over-printing, on the order of hundreds of microns (c, scale bars = 2 mm). For PCL, there is essentially no under-printing, but over-printing occurs on the order of millimters (g,k). The dramatic over-printing is attributed to the large PCL particle size, and shows little difference between smoothed and unsmoothed PCL. Deviation histograms quantify the deviation between scan and model for 160 slices through 3 lattices (nylon, d) and 460 slices through 4 lattices (PCL; h,l). For nylon, >60% of scanned points overlap the model and <5% of scanned points differ from the model by >200 μm. In contrast, only ~20% of scanned PCL points can overlap the reduced diamond lattice model, with nearly 50% of points falling between 1–3 mm away from the model.

To analyze how accurately complex geometries are reproduced in OpenSLS, we performed a dimensional fidelity analysis of nylon and PCL diamond lattices (Fig 4). For nylon, a representative slice shows that a μCT virtual cross-section through the sintered part closely reflects the geometry of the original CAD model (Fig 4A). A heatmap of the deviations between model and μCT scan show substantial overlap, with minor protrusions and gaps in the sintered part (Fig 4C). Extending this analysis to 150 slices through 3 nylon lattices showed that >60% of all pixels containing scanned or model geometry were overlapping. Out of the pixels that did not overlap, <5% of all overprint and underprint extended past 200 μm (Fig 4D).

For PCL diamond lattices, representative cross-sections highlight the major overprinting that occurs due to PCL grain size (Fig 4E, 4F, 4I and 4J). The edges of individual PCL grains are visible protruding from filament cross-sections in sintered PCL (Fig 4F). For smoothed PCL, the degree of overprint appears similar, but individual PCL particles are no longer distinguishable (Fig 4J). Deviation heatmaps for sintered and vapor-smoothed PCL indicate that the scans may be off-center with respect to the model geometry (Fig 4G and 4K). This is probably due to imperfect alignment of scan and model STL files, which is challenging due to the complexity of the geometries. It is important to note that alignment is performed on the entire 3D structure, such that the overall optimal alignment may not be ideal for a particular slice. Slight differences are observed in the deviation histograms of sintered and vapor-smoot.hed PCL, indicating that vapor-smoothed PCL has less overlap and more >1 mm deviations than sintered, unsmoothed PCL (Fig 4H and 4L). Based on the asymmetry of deviations in the heatmap, we propose that these differences may be attributed to imperfect alignment.

**Fig 5:**
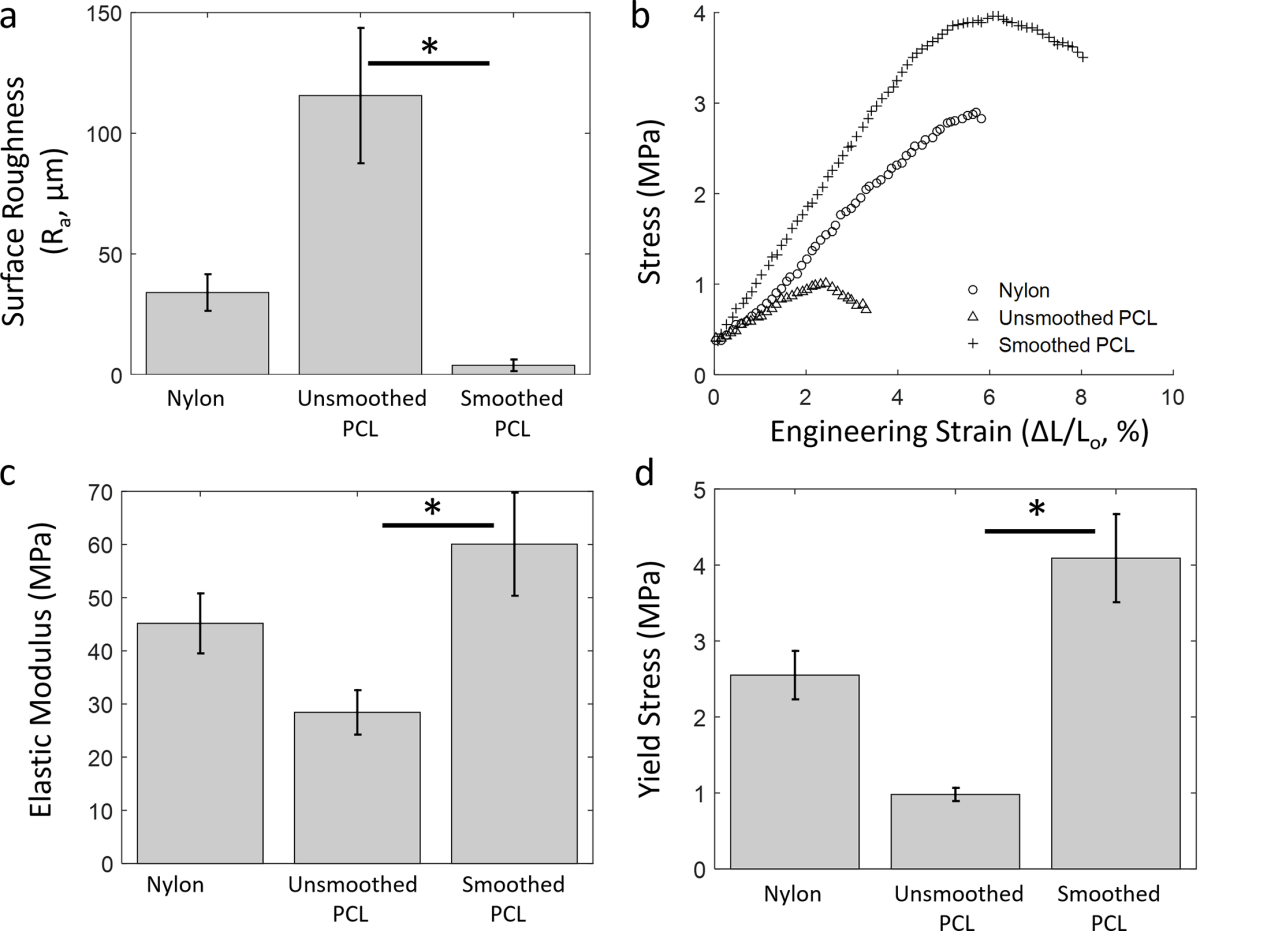
Surface roughness and mechanical testing of sintered nylon and PCL. a) Surface roughness (R_a_) of PCL decreases nearly 30-fold as a result of vapor-smoothing. R_a_ for both nylon and unsmoothed PCL is on the order of magnitude of the particle size. b) Representative stress-strain curves for uniaxial compression testing of nylon and PCL macroporous cylinders (geometry shown in S3 Fig). All three materials demonstrate linear deformation until failure. c) The elastic modulus of PCL is doubled as a result of vapor-smoothing and d) the yield stress increases four-fold (n = 5 cylinders). The significantly improved mechanics of smoothed PCL make it a superior candidate material for bone tissue engineering. * denotes p < 0.01 using Student’s T-test. Plots represent mean ± SD.

An important metric for the quality of laser sintered parts is the surface roughness, which influences not only the aesthetic appearance of the part, but also its ability to interface properly with other parts. Additionally, surface texture and topography are of particular importance when considering surfaces on which cells will be seeded [71–73]. Sintered nylon parts were found to have average surface roughness (R_a_) 34.0 ± 7.6 μm, a value consistent with both the particle size of nylon and prior determinations of R_a_ for sintered nylon using commercial SLS systems (Fig 5A) [74]. Nylon surface finish could be improved through abrasive post-processing, as has been demonstrated previously [74]. R_a_ for unsmoothed PCL was measured as 115.6 ± 28.0 μm, reflecting the visibly rough texture of sintered PCL parts. After vapor-smoothing, however, R_a_ for PCL decreased to 3.9 ± 2.4 μm, qualitatively visible in Fig 3D and 3E. In fact, 3.9 μm is likely an overestimate for the surface roughness of smoothed PCL which arises from slight curvature at the ends of the analyzed edges; SEM imaging (Fig 3E) indicates that the average surface roughness is actually sub-micron. As discussed below, the significant reduction in surface roughness enabled cells seeded on smoothed PCL to more consistently exhibit characteristic morphology.

Like many additive processes, the time required to build a part using SLS scales with both size and complexity of the 3D model. For example, a solid cube might require less time to pattern than a cube of the same dimensions containing macropores, despite the greater volume of material used in the solid cube. For any part, it is always more efficient to sinter multiple copies at once than single copies sequentially so that the process of creating new layers occurs only once instead of for each model. Similarly, it is least efficient to sinter models whose long axis is oriented in the z-direction because adding new layers is generally more time-intensive than adding geometry to existing layers. Additionally, the efficiency is material dependent; different laser scan speeds are optimal for individual materials, contributing to disparities in build time for the same geometry. For all these reasons, quantifying the efficiency of OpenSLS is difficult and a straightforward relationship between model volume and build time does not exist. In the absence of a predictive relationship to determine build time, the times required to build geometries used in this study are shown (Table 2).

**Table 2:** Build times for 3D models using OpenSLS. Cube, Cube Dimensional Accuracy Model (1.04 cm^3^); ASTM Cylinder, ASTM Cylinder with Macropores (0.54 cm^3^, see S3 Fig); Diamond, Diamond Lattice (0.90 cm^3^, see Fig 2); Reduced Diamond, Reduced Diamond Lattice Model (0.21 cm^3^, see Fig 3).

Nylon			PCL		
Geometry	Number of Copies	Build Time (mins)	Geometry	Number of Copies	Build Time (mins)
**Cube**	1	33	**Cube**	1	16
**Cube**	4	75	**Cube**	4	37
**Cube**	9	143	**Cube**	9	71
**ASTM Cylinder**	1	62	**ASTM Cylinder**	1	29
**ASTM Cylinder**	4	125	**ASTM Cylinder**	4	52
**ASTM Cylinder**	16	374	**ASTM Cylinder**	16	145
**Diamond Lattice**	1	60	**Reduced Diamond**	1	37
**Diamond Lattice**	2	88	**Reduced Diamond**	2	56
**Diamond Lattice**	4	146	**Reduced Diamond**	4	95

### Mechanical Testing

For structures produced via additive manufacturing, mechanical properties vary between geometries and may be markedly different from the bulk material properties. Uniaxial compression testing of nylon and PCL yielded linear stress-strain plots until failure, caused by buckling of vertical filaments (Fig 5B). Eshraghi and Das [36] comprehensively studied the mechanics of macroporous PCL lattices prepared via SLS on a commercial system. Here, we mechanically tested the same geometry–a modification to ASTM standard 695-02a containing macropores in 3 dimensions–enabling a relatively direct comparison between the mechanics of structures sintered on a commercial system versus OpenSLS (S3 Fig). (Eshraghi and Das used 125 μm raw PCL, a ~25% reduction in size compared to our CAPA 6506 (S1 Fig)).

For elastic modulus, Eshraghi measured E = 14.9 MPa and we measured 28.4 ± 4.2 MPa and 60.1 ± 9.7 MPa for sintered and vapor-smoothed PCL, respectively (n = 5, Fig 5C) [36]. Williams *et al*. measured the compressive modulus for macroporous sintered PCL as 52–68 MPa, in reasonably good agreement with our findings [31]. The slightly higher values we measured could be explained by a larger cross-sectional area caused by PCL overprinting (see Fig 4). We found a similar trend for the yield strength of sintered (0.98 ± 0.1 MPa) and vapor-smoothed (4.1 ± 0.8 MPa) PCL, which are slightly and moderately greater than Eshraghi’s measurement of 0.60 MPa (Fig 5D). Thus, vapor-smoothing makes PCL better suited for bone tissue engineering based on its ability to withstand greater compressive loading. Material failure occurred at 2.5 ± 0.39% for sintered PCL, in excellent agreement with 2.7% found by Eshraghi. Vapor-smoothing increased the strain at yield to 8.4 ± 2.1%, demonstrating that vapor-smoothed PCL is both stronger and more deformable than sintered, unsmoothed PCL. Overall, we have demonstrated that PCL structures laser sintered using OpenSLS closely match the mechanical properties of those sintered using commercial systems. Furthermore, we have established a key advantage for using vapor-smoothed PCL in bone tissue engineering by showing improved mechanical properties compared to sintered, unsmoothed PCL.

Macroporous nylon specimens also underwent compression testing. For nylon, fitting the linear region of the stress-strain curve for 8 samples yielded an average elastic modulus of 45.2 ± 5.6 MPa (Fig 5C), yield stress of 2.5 ± 0.3 MPa (Fig 5D), and strain at yield of 5.5 ± 0.7%. No data could be found in the literature for compression testing of macroporous nylon cylinders, which are expected to have mechanical properties uniquely associated with their geometry. However, we did note that the recorded elastic modulus for macroporous nylon cylinders is an order of magnitude below the reported stiffness of solid nylon [57]. This magnitude of this difference is similar to the difference found previously between macroporous PCL cylinders and solid PCL cylinders [36].

### Sacrificial templating of PCL to form fluidic networks

**Fig 6:**
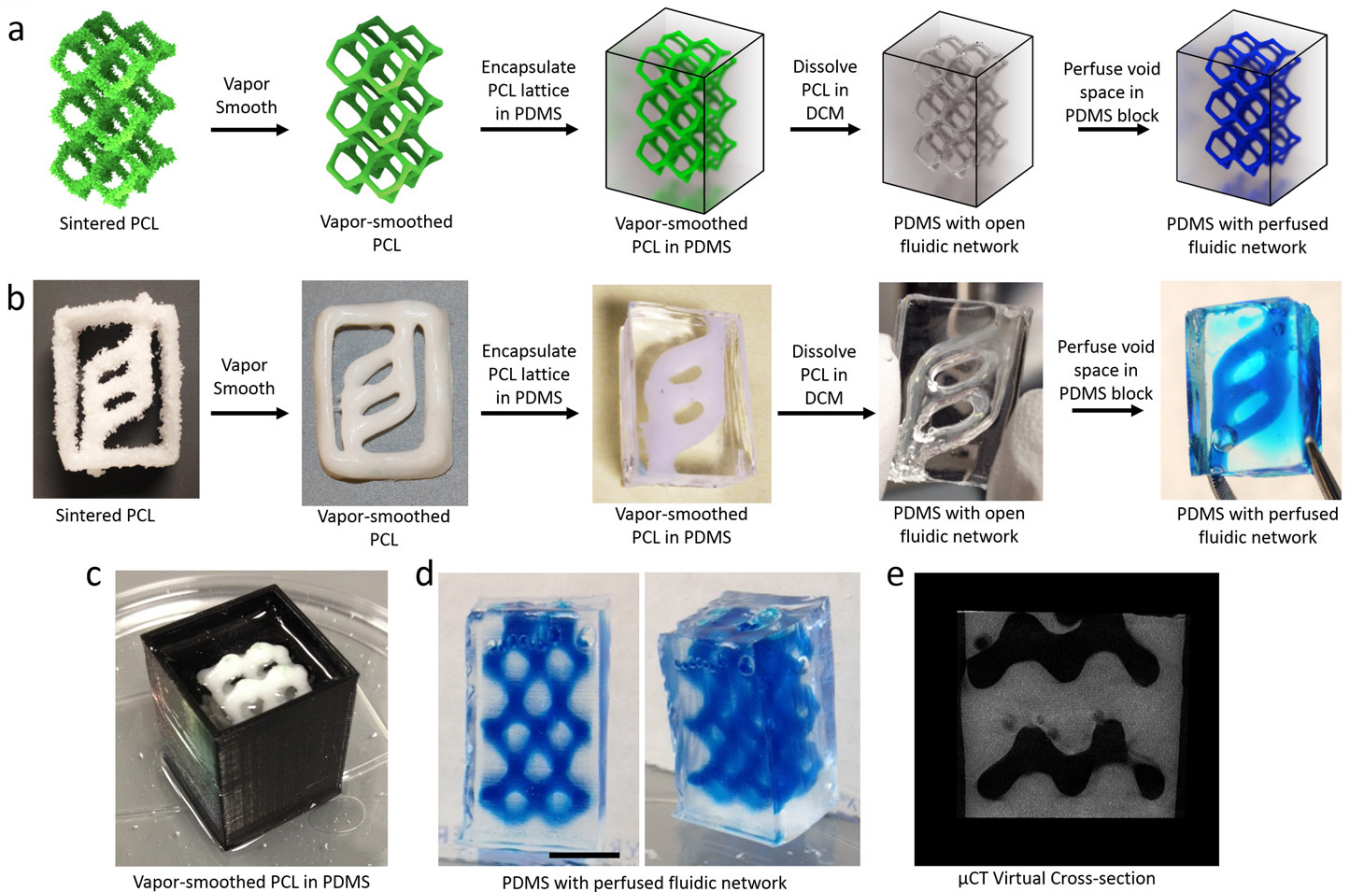
Fluidic networks templated by sacrificial PCL structures. a) Schematic for a workflow which begins with a sintered PCL structure and yields the corresponding fluidic network as void space in a PDMS slab. The original PCL structure is vapor smoothed before encapsulation in a block of PDMS. The smoothed PCL is dissolved out of the cured PDMS using DCM, leaving a fluidic network that retains the architecture of the original structure. b) The workflow schematized in (a) is demonstrated with a simple ladder geometry. The inlet and outlet allow perfusion and continuous flow through the network. c-e) Sacrificial templating of the reduced diamond lattice model (Fig 3) resulted in the formation of a complex, interconnected fluidic network in PDMS. Perfusion with blue dye (d, scale bar = 1 cm) highlights the interconnectivity of the void space and a virtual cross-section through a μCT scan (e) demonstrates fluidic channels retaining the original structure’s geometry (artifacts are present due to bubbles trapped in PDMS).

As fluidic networks are increasingly used in microfluidic chips and diagnostic devices, it is valuable to investigate techniques by which such networks can be assembled via additive manufacturing. We used sintered, vapor-smoothed PCL structures to sacrificially template fluidic networks in PDMS. In sacrificial templating, a template structure is fabricated in a temporary material and encased in a second bulk material. Selective removal of the temporary material patterns the template structure as void space within the bulk material [75,76]. When sintered, vapor-smoothed PCL was encapsulated in PDMS, then dissolved out by immersion in DCM, the geometry of the sintered PCL was retained as void space within the PDMS (schematized in Fig 6A). This method can produce a wide range of perfusable fluidic networks within monolithic PDMS slabs. For example, sacrificial templating of a simple ladder geometry yielded open channels in place of the PCL filaments as well as an inlet and outlet for perfusion (Fig 6B). This fluidic network is reminiscent of a primitive vascular network model that we introduced previously [12].

We also templated a diamond lattice geometry to generate a complex, interconnected fluidic network in PDMS (Fig 6C). Perfusion illustrated that this network is fully interconnected (Fig 6D). Scanning the patterned PDMS with μCT showed that the template geometry was well preserved when PCL was selectively removed (Fig 6E). The templating of diverse perfusable fluidic networks offers a method for creating model vascular architectures for flow analysis and for fabricating fluidic devices and flow phantoms with complex channel arrangements [77,78]. This method is similar to one recently published, wherein 3D-printed plastic filament was used as a sacrificial template for microfluidic networks [79]. Using SLS to generate sacrificial templates (rather than extrusion) expands this technique by enabling fabrication of fluidic networks whose architecture cannot be printed via extrusion 3DP.

### Morphology, viability, and osteogenic differentiation of human mesenchymal stem cells on sintered and vapor-smoothed PCL

**Fig 7:**
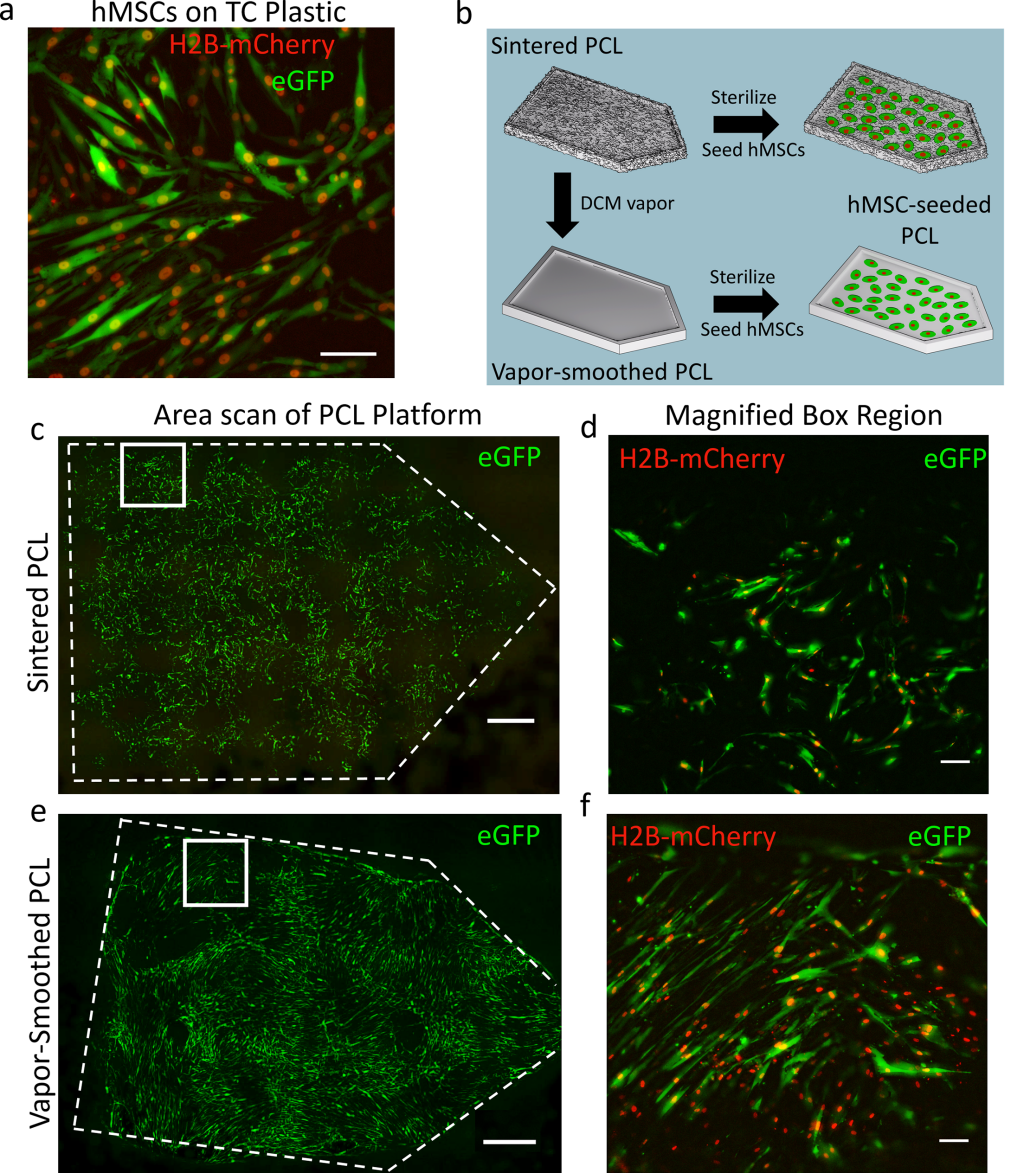
Morphology of hMSCs seeded on PCL platforms fabricated with OpenSLS. a) When hMSCs, constitutively expressing GFP (cytoplasm) and H2B-mCherry (nucleus), were seeded on tissue culture plastic (TCP), cells exhibited elongated, spindle-like morphology and alignment of neighboring cells. b) Schematic depicting seeding of GFP/H2B-mCherry-labeled hMSCs onto sintered (unsmoothed) as well as vapor-smoothed PCL platforms. c) After 10 days in culture, hMSCs populated the surface of the sintered PCL platform as a sparse monolayer (scale bar = 1000 μm). d) hMSCs grown on sintered PCL exhibit a spindle-like morphology but are not spread out or aligned to the degree observed on TCP (scale bar = 100 μm). e) On sintered, vapor-smoothed PCL, a dense monolayer of hMSCs was observed with regions of local cell alignment (scale bar = 1000 μm). f) In contrast to hMSCs grown on sintered, unsmoothed PCL, those seeded on vapor-smoothed PCL exhibited highly elongated spindle-like morphology characteristic of hMSC culture on TCP (scale bar = 100 μm). Gamma correction was used to improve visualization of cells.

The biocompatibility of PCL has been validated previously through use in drug delivery devices [80,81], tissue engineering scaffolds [82,83], and sutures [84]. Indeed, the FDA has approved PCL for implantation inside the body as well as in several drug delivery devices [85]. Here, we sought to verify that PCL is suitable for cell seeding after undergoing sintering and smoothing with DCM. When human mesenchymal stem cells (hMSCs) were seeded onto sintered PCL platforms, we observed adhesion of cells in a monolayer on both unsmoothed and vapor-smoothed surfaces after 10 days in culture. Qualitatively, cells appeared to adhere at higher densities on vapor-smoothed surfaces owing to the contiguous surface (Fig 7C and 7E). Cell morphology was also influenced by vapor smoothing. On sintered PCL, minimal elongation of cells was observed in comparison to the extremely elongated, spindle-like morphology visible on vapor-smoothed PCL (Fig 7D and 7E). This elongated morphology, in combination with the local alignment of neighboring cells, gave hMSCs seeded on vapor-smoothed PCL a similar appearance to hMSCs grown on tissue culture plastic (Fig 7A). Similar morphology has also been observed previously after Salerno and colleagues seeded hMSCs onto a PCL scaffold with porosity introduced via gas foaming [86]. Since the surface topography of vapor-smoothed PCL encourages a physiologically relevant hMSC morphology, it may be a superior material for bone tissue engineering compared to sintered, unsmoothed PCL.

**Fig 8:**
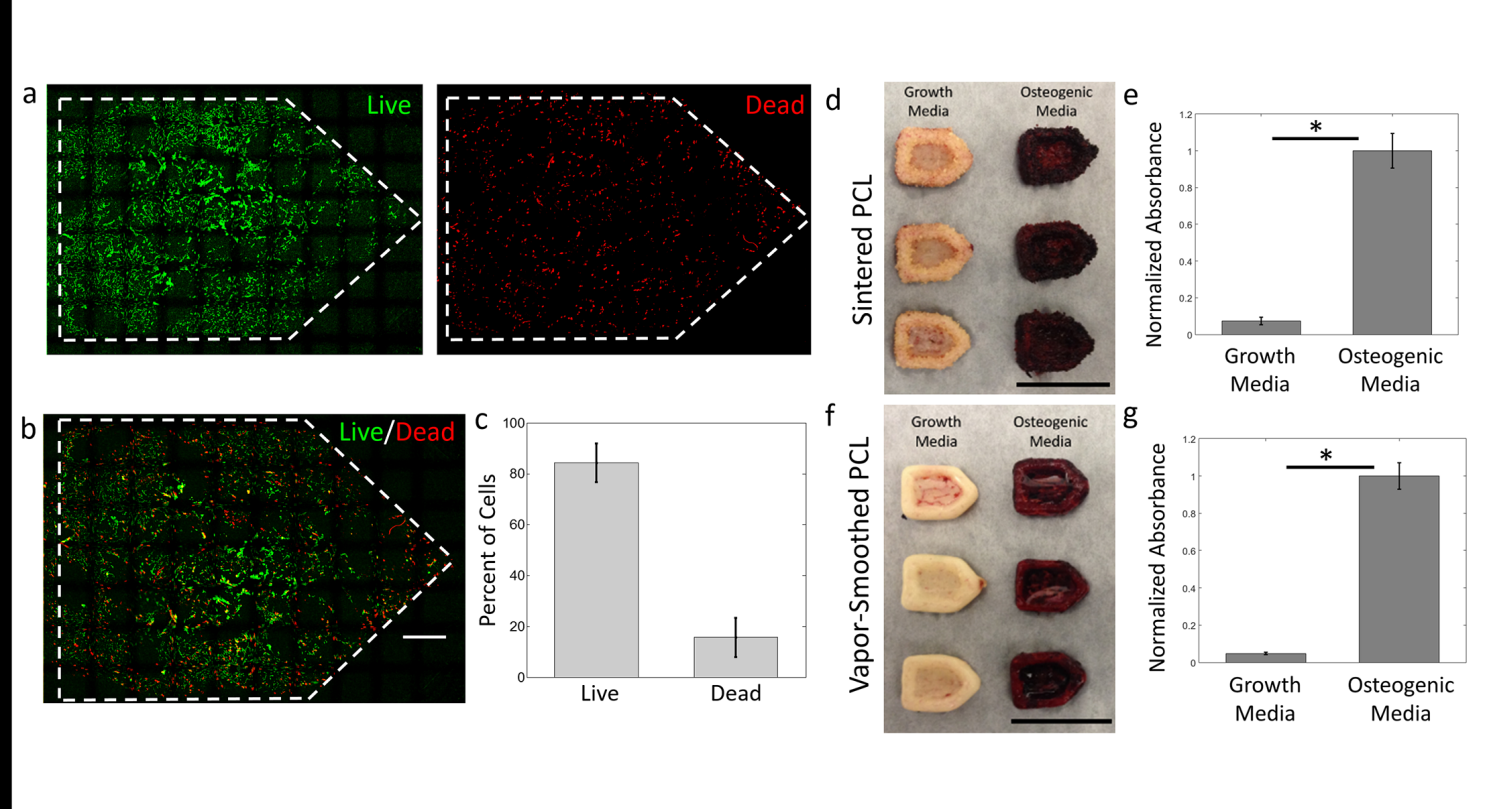
Survival and osteogenic differentiation of hMSCs on PCL fabricated via OpenSLS. a,b) Live and dead channels for live/dead staining of hMSCs on vapor-smoothed PCL platforms show a majority live cells and a generally homogeneous distribution of dead cells among live cells. Gamma correction was used to improve visualization of cells. c) Quantification of live and dead hMSCs from three separate PCL platforms showed that 84 ± 7% of adhered cells were alive. d) Gross images of sintered PCL after 32 days show intense staining on platforms seeded with hMSCs incubated in osteogenic media (osteogenic platforms), indicating the presence of calcium deposits characteristic of early osteoblasts. e) Quantification of alizarin red absorbance shows a nearly 15-fold increase in staining on osteogenic platforms compared to those cultured in growth media. f,g) The same intense staining of osteogenic PCL platforms was observed when the PCL was vapor smoothed prior to seeding of hMSCs. Scale bars = 1 cm. * denotes p < 0.01 using Student’s T-test. Plots represent mean ± SD.

We further validated the biocompatibility of sintered PCL platforms through live/dead staining of seeded hMSCs. Over three experiments, we found that approximately 85% of cells were viable 1 day after seeding, demonstrating that losses in viability are minimal (Fig 8A–8C). These results are consistent with viability assays in the literature conducted using hMSCs on PCL [87]. We were unable to appropriately image or quantify hMSC viability on unsmoothed sintered PCL platforms due to their extremely rough surface topography.

Culture of hMSCs on sintered PCL is relevant for bone tissue engineering insofar as the hMSCs can be reliably differentiated along an osteogenic lineage. Indeed, differentiation of hMSC populations into osteoblasts has emerged as a promising strategy for encouraging mineralization and maturation of engineered bone [88]. We verified that PCL platforms fabricated using OpenSLS are compatible with this approach by osteogenically differentiating hMSCs seeded on these platforms. Using Alizarin Red S staining, we measured a 15-fold increase in calcium deposits on PCL platforms which were incubated 32 days in osteogenic media relative to platforms maintained in growth media (Fig 8D–8F). These calcium deposits, a hallmark of early osteoblasts, demonstrate that under appropriate conditions, hMSCs will undergo osteogenic differentiation on sintered PCL platforms fabricated using OpenSLS. Overall, our results for hMSC viability, morphology, and differentiation indicate that structures fabricated via OpenSLS are well-suited for cell studies, including cell studies relevant to engineering bone.

## Conclusions

We demonstrated an open-source SLS system that can be assembled for under $10,000 USD, representing a 10- to 50-fold decrease in cost compared to commercially available systems. With this system, nylon structures can be fabricated with sub-millimeter features and dramatic overhangs. The potential for sintering relevant materials for tissue engineering was demonstrated through sintering of polycaprolactone, which was compatible with hMSC viability, characteristic morphology, and osteogenic differentiation. OpenSLS could serve the scientific community as an accessible platform for fabrication of structures composed of a wide range of materials, including non-traditional materials not supported by commercial SLS suppliers. Overall, OpenSLS is a powerful and low-cost tool that makes additive manufacturing via laser sintering feasible and cost-effective for scientific laboratories.

## Supporting Information

S1 File Detailed information on the hardware and software configuration for OpenSLS. (PDF)

S1 Bill of Materials A summary of the components needed to build OpenSLS and the prices and vendor information for each component. (XLSX)

S1 Fig Characterization and sizing of powdered nylon and polycaprolactone. a,c) SEM micrographs of nylon and PCL powder at 35x demonstrate that the respective raw materials differ in size by approximately an entire order of magnitude (scale bars = 1mm). b,d) High-magnification SEM micrographs of nylon and PCL show that both particles have rough surfaces at this scale, but nylon particles are more spherical and less irregular (scale bars = 50μm). e) Particle size distributions were determined for nylon using SEM (n = 1007) and for PCL using optical images (n = 1168). Sizing data from optical microscopy was validated by comparing particle measurements with measurements of the same particles using SEM (S3 Fig). The size distributions were quantified as minimum Feret diameter (left) and maximum Feret diameter (right). The minimum Feret diameter data is reasonably consistent with the technical data provided by the suppliers, and is likely a closer approximation for sieving- or diffraction- based sizing. The shift in histograms from minimum to maximum Feret diameter is far more dramatic for PCL than Nylon, further demonstrating the relative roundness of nylon and the irregularity of PCL particles.
(TIF)

S2 Fig Sizing data from optical microscopy validated by comparison to SEM measurements. The same sample of PCL was imaged on an optical microscope, then sputter-coated and measured on SEM. Lengths measured with ImageJ from the optical microscope image (left; scale bar = 1000μm) were compared to the corresponding length measured on the SEM micrograph (right; scale bar = 1000 μm). On average, the difference between the size measurements was 3.8%, validating the use of optical microscope images for particle sizing.
(TIF)

S3 Fig Modified ASTM Geometry for Mechanical Testing. a) Rendered CAD model of a macroporous modification to ASTM Standard D695-02a geometry (cylinder, 25.4mm height, 12.7 mm diameter). This modification was introduced previously by Eshraghi and Das, see [36]. b) Modified ASTM lattice depicted in (a) was sintered from nylon and underwent uniaxial compression testing. The failure point for the lattice is evident from the distorted structure. c) Modified ASTM lattice (a) was sintered in PCL and vapor smoothed. PCL is much less brittle than nylon, so the failure point from compression is not readily visible. Scale bars = 5mm.
(TIF)

S1 Video Laser sintering of nylon diagrid model using OpenSLS. (MP4)

S2 Video Time-lapse video of vapor smoothing sintered PCL using DCM vapor. (MP4)
