# Supplementary material for: Open-Source Selective Laser Sintering (OpenSLS) of Nylon and Biocompatible Polycaprolactone
Source: PLoS One. 2016 Feb 3;11(2):e0147399. doi: 10.1371/journal.pone.0147399 (PMC4739701; doi:10.1371/journal.pone.0147399)
Supplement: S1 File — (PDF) [file pone.0147399.s001.pdf]

Supporting Information for

Open-source Selective Laser Sintering (OpenSLS) of Nylon and Biocompatible Polycaprolactone

Ian S. Kinstlinger*, Andreas Bastian*, Samantha J. Paulsen, Daniel H. Hwang, Anderson Ta, David R. Yalacki, Tim Schmidt, and Jordan S. Miller

Department of Bioengineering, Rice University, Houston, Texas, USA

Materials and Configuration of OpenSLS

Bill of Materials

The bill of materials for OpenSLS is provided (S1 Bill of Materials), and can also be found on the OpenSLS github repository (https://github.com/MillerLabFTW/OpenSLS). Future updates to the design files or firmware for OpenSLS will be made available through this repository. The exact dimensions of components may depend on the laser cutter that is used. For example, the dimensions for the laser-cut powder module might require adjustment.

Wiring

Caution: extreme care should be taken when modifying the wiring of the laser and its power supply. Laser power supplies operate with voltages on the order of tens of kilovolts and may hold charge for some time after being powered down.

Figure A shows the wiring diagram used for OpenSLS. The motors illustrated to the left of the RAMBo board are the NEMA17 motors that are part of the powder handing module, while the motor drivers shown to the right drive the native stepper motors inside the laser cutter. Matching the ground planes for the laser power supply, stepper drivers, and RAMBo board is critical.

Firmware

OpenSLS uses a customized version of the widely used open-source Marlin for Arduino, available through the github repository. We have configured the firmware to disable features irrelevant to OpenSLS (e.g. temperature control, endstop detection, servo support). We added functionalities corresponding to features of SLS, and extended the firmware's G-code machine language to accommodate the new functions (Table A).

Initially, laser firing and extinguishing were implemented in Marlin (Marlin_main.cpp), in the G-code processing routine. Laser firing and extinguishing were functional, but their timing relative to other machine operations wasn't satisfactory. Laser intensity control was moved to a 16 bit timer, chosen automatically based on the setting of LASER_INTENSITY_PIN in pins.h. Laser firing and extinguishing execution were moved to the stepper driver interrupt handler, where G-codes are ultimately evaluated, requiring the addition of laser-specific parameters to the firmware's command buffer. A counter tracking laser firing time was added and is stored in the microcontroller's EEPROM to maintain persistence across power cycles. An L axis was added to Marlin's base 5-axis configuration (X, Y, Z, E0 and E1 axes) to facilitate calculation of laser pulses per millimeter. A runtime-configurable laser debugging mode with verbose serial output was added. Laser-related menu items were added for the “LCD Smart Controller” and “Full Graphic LCD Smart Controller”. Lastly, laser firing, extinguishing, and maintanance functions were consolidated in laser.h and laser.cpp

The implementation permits laser operation in three distinct firing modes:

1) Continuous mode: the laser is turned on and remains on at the selected intensity until it receives a G-code instructing it to turn off. Continuous mode firing has been used for all OpenSLS firing commands at this time.

2) Pulsed mode: the laser fires punctuated bursts at intervals matching P: PULSES_PER_MM, each lasting for L: DURATION (in μs). The values of P and L are specified within the laser firing G-code (see Table A) so that the timing and duration of the laser pulses can be easily added to the firmware's command buffer with the laser firing command. This allows us the laser to be fired and extinguished from the inner loop of the stepper driver interrupt handler, enabling microstep-accurate positioning and accurate timing of laser pulses.

3) Raster mode: a special variation of pulsed mode which accepts an intensity value for each pulse in a variable-length horizontal line of evenly spaced pulses, with configurable pulse offsets in X and Y axes, arbitrary line-advance, and selectable left or right rastering. Like pulsed mode, timing is performed in the stepper driver interrupt handler, and the information necessary for many pulses is contained in a single command, allowing for very precise high-speed control. A possible future development within this mode is support for rastering along an arbitrary line in 3D space.

We also implemented several new compile-time configuration options in the firmware:

LASER_CONTROL: When set to 1, the LASER_FIRE pin supplies a PWM signal at frequency specified by LASER_PWM in Configuration.h. The duty cycle of the wave is adjusted to control intensity, logic level LOW is set when off. This configuration works well for controlling common laser diodes. When set to 2, the LASER_FIRE pin supplies a logic level signal HIGH for fire, LOW for extinguish. The LASER_INTENSITY pin supplies a PWM signal at frequency specified by LASER_PWM in Configuration.h, and duty cycle of the wave is adjusted to control intensity.

LASER_FIRE_G1: G0 moves the laser to a specified set of coordinates <u>without</u> firing, a G1 command moves the laser to the specified coordinates <u>while</u> firing.

LASER_FIRE_SPINDLE: M3 turns the laser on in place, without requiring it to move. M5 turns the laser off in place.

LASER_FIRE_E: Any movement in the E axis (which represents the extruder when controlling a 3D printer) fires the laser. In this way, the laser can make use of unmodified G-code generated for 3D printers. Note: this mode is not recommended in OpenSLS because extruder axes are already controlling the power reservoir and build platform motors.

Table A shows the specific G-codes added in our firmware and their functions. An excellent resource for the standard G-code language used in this firmware to control OpenSLS can be found on the RepRap wiki (reprap.org/wiki/G-code).

G-code	Function	Notes
G1	Move while laser firing	In unmodified Marlin, G0 and G1 refer to the same action.
G0	Move while laser off	
M3	Laser on (fire)	A G1 command will fire the laser on its own and does not need to be preceded by an M3 command
M5	Laser off (extinguish)	
M649	Modify laser settings	This command takes the format “M649 S# L# P# D# B#”<br>S: Intensity of laser firing (0-100.0%)<br>L: Duration of firing (in microseconds)<br>P: Pulse per mm (in pulse mode)<br>D: Diagnostic mode (0 = off; 1 = on)<br>B: Laser firing mode (0 = continuous, 1 = pulsed, 2 = raster)<br>This command can be used to set laser settings before starting to sinter, or to adjust settings during sintering.

Table A. G-codes unique to OpenSLS and their functions

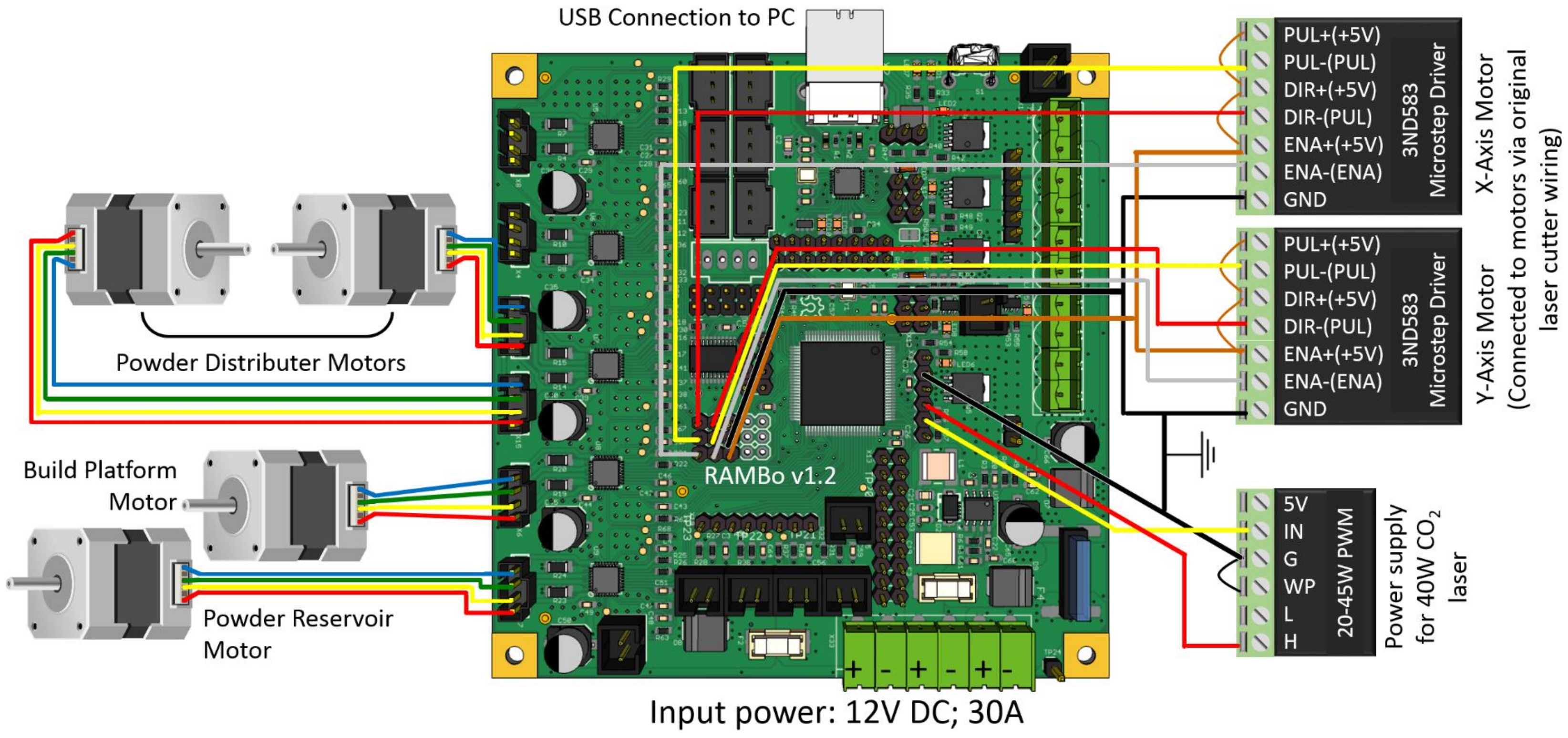

Figure A. Wiring diagram for OpenSLS. X and Y axis motor controls used Leadshine microstep drivers already onboard the laser cutter. The 40W laser power supply replaced the 80W supply natively integrated with the laser cutter. Plow motors (RAMBo Z-axis) and powder-handing piston motors (RAMBo E0- and E1-axes) are standard NEMA17 stepper motors. OpenSLS's custom hardware configuration requires a modified version of typical open-source 3DP firmware which re-maps X and Y control to the motor extension pins and implements laser control via the PWM pins.

Slicing Settings in Slic3r Software

The settings used for laser sintering with OpenSLS differ considerably from the typical defaults used for melt extrusion printing. A configuration file for the open-source Slic3r software (slic3r.org) is included on the OpenSLS github repository, and the settings are indicated in Table B. An important adjustment necessary in preparing geometries for OpenSLS involves scaling the model along its z-axis. Slic3r settings do not allow a layer height taller than the nozzle diameter, so we treat the layer height as 0.05mm in Slic3r. In actuality, the layer height is 0.15 (nylon) or 0.30mm (PCL). To compensate, the geometry needs to be scaled down in z by the scaling factor between Slic3r layer height and actual layer height. For example, a model sliced with layer height 0.05mm and sintered with layer height 0.15 should be shrunk by a factor of 3 in the z-axis (Netfabb Basic, netfabb.com).

Parameter	Value		Parameter	Value
Layer height	0.05 mm		Infill density	Variable – we often used 20%
First layer height	0.05 mm		Fill pattern	Concentric
Perimeters (min)	0		Infill every 1 layers	
Horizontal Shells	0 (Top & Bottom)		Solid infill every 0 layers	
Extra perimeters if needed	No		Support material	Disable
Detect thin walls	Yes		Skirt	0 loops
Detect bridging perimeters	Yes		Brim width	0 mm
Speed: perimeters	30 mm/s		Bed Size	X: 70 Y: 70 mm
Speed: infill	60 mm/s		Bed Center	X: 35 Y: 35 mm
Speed: travel	130 mm/s		Nozzle Diameter	0.075 mm
First layer speed	50%			

Table B. Parameters used for slicing for nylon in Slic3r version 1.1.7. A .ini configuration file with these parameters is available on the OpenSLS github.

G-code Munging

The G-code output from slicing an STL model is meant to be interpreted by a melt extrusion printer, not SLS. Thus, the G-code must be modified so that Z changes invoke the addition of a new powder layer and XY motion is assigned the appropriate laser firing behavior. We performed munging (data editing) of the G-code with a custom PHP script that is available on the OpenSLS Github repository. The PHP script is run from the command line as follows (example in Windows operating system):

```
> cd C:\TargetDirectory
> "C:\...\PHP\php.exe" "C:\...\Munge\munge.php" –i "C:\...\file.gcode"
```

This results in the creation of the modified G-code in TargetDirectory with the name output.gcode. An example of G-code before and after munging is shown in Box A.

```
;Original Gcode file from Slic3r
G1 X24.000 Y43.250
; XY move without extrude
;not patterning material
G1 X23.750 Y27.000 E0.09499
; XY move with extrude
;patterning material
G1 Z0.150 F7800.000
; Z move
G1 X24.000 Y26.750 F600.000
; XY move without extrude
```

```
;Modified Gcode file after munge
G0 X24.000 Y43.250
; G1 becomes G0 so laser does not fire
G1 X23.750 Y27.000
; G1 stays G1 so laser fires, extrude removed
; Z move is replaced by new powder layer
T0 ;select E0 axis (build platform motor)
G0 E0.15 F200 ; move build piston down 0.15mm
T1 ;select E1 axis (reservoir motor)
G0 E0.45 F200 ; move reservoir up 0.45mm
G0 X-240 Y38 F8000 ; move laser away
G0 Z170 F1500 ; translate the powder distributor
; to move powder from reservoir to build platform
G0 Z20 F1500 ; return distributor to origin
G0 X24.000 Y26.750 F600.000
; G1 becomes G0 so laser does not fire
```

Box A. Sample G-code before and after munging for OpenSLS compatibility
