# Supplementary figures and images for: Open-Source Selective Laser Sintering (OpenSLS) of Nylon and Biocompatible Polycaprolactone
Source: PLoS One. 2016 Feb 3;11(2):e0147399. doi: 10.1371/journal.pone.0147399 (PMC4739701; doi:10.1371/journal.pone.0147399)

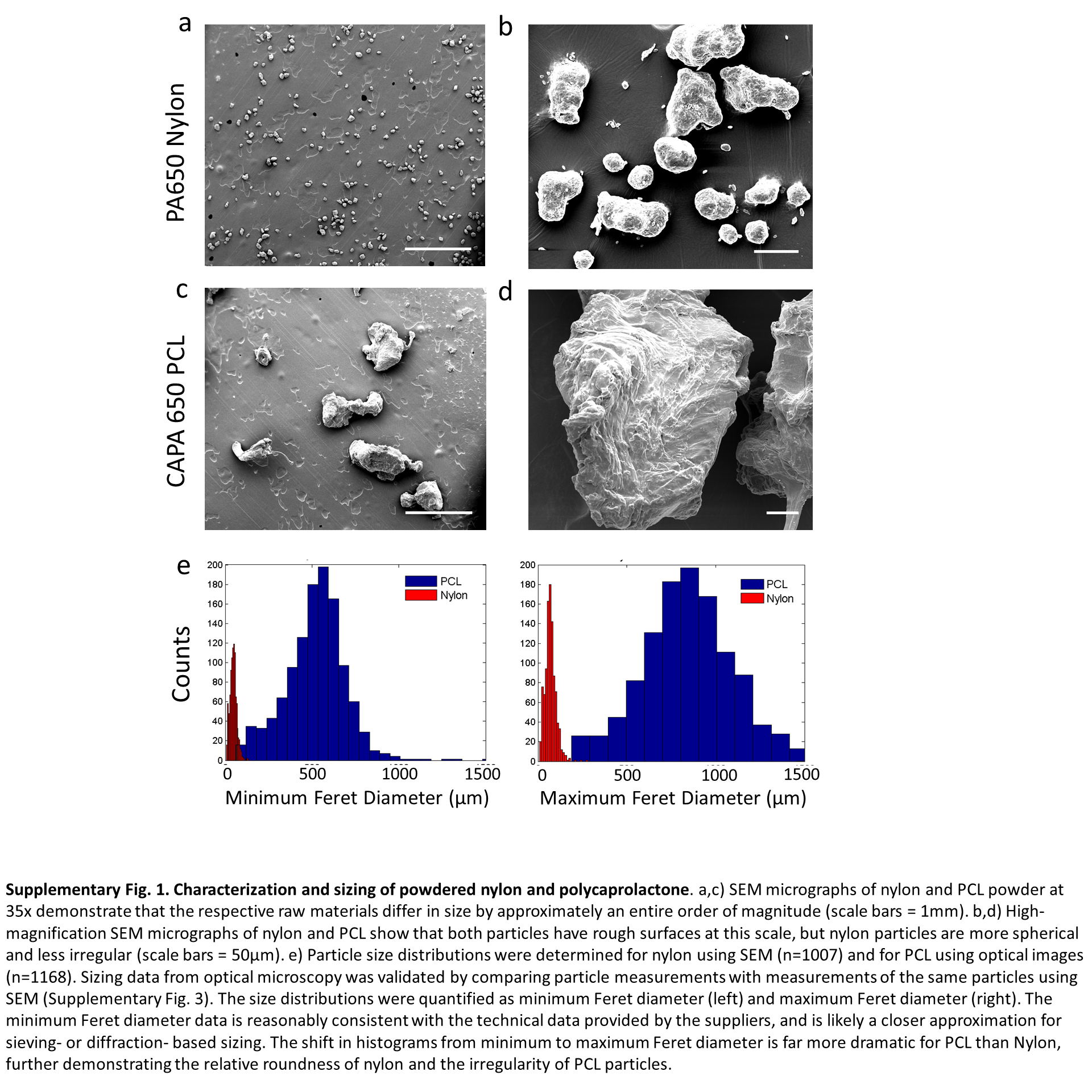

Supplement: S1 Fig — a,c) SEM micrographs of nylon and PCL powder at 35x demonstrate that the respective raw materials differ in size by approximately an entire order of magnitude (scale bars = 1mm). b,d) High-magnification SEM micrographs of nylon and PCL show that both particles have rough surfaces at this scale, but nylon particles are more spherical and less irregular (scale bars = 50μm). e) Particle size distributions were determined for nylon using SEM (n = 1007) and for PCL using optical images (n = 1168). Sizing data from optical microscopy was validated by comparing particle measurements with measurements of the same particles using SEM (S3 Fig). The size distributions were quantified as minimum Feret diameter (left) and maximum Feret diameter (right). The minimum Feret diameter data is reasonably consistent with the technical data provided by the suppliers, and is likely a closer approximation for sieving- or diffraction- based sizing. The shift in histograms from minimum to maximum Feret diameter is far more dramatic for PCL than Nylon, further demonstrating the relative roundness of nylon and the irregularity of PCL particles. (TIF) [file pone.0147399.s003.tif]

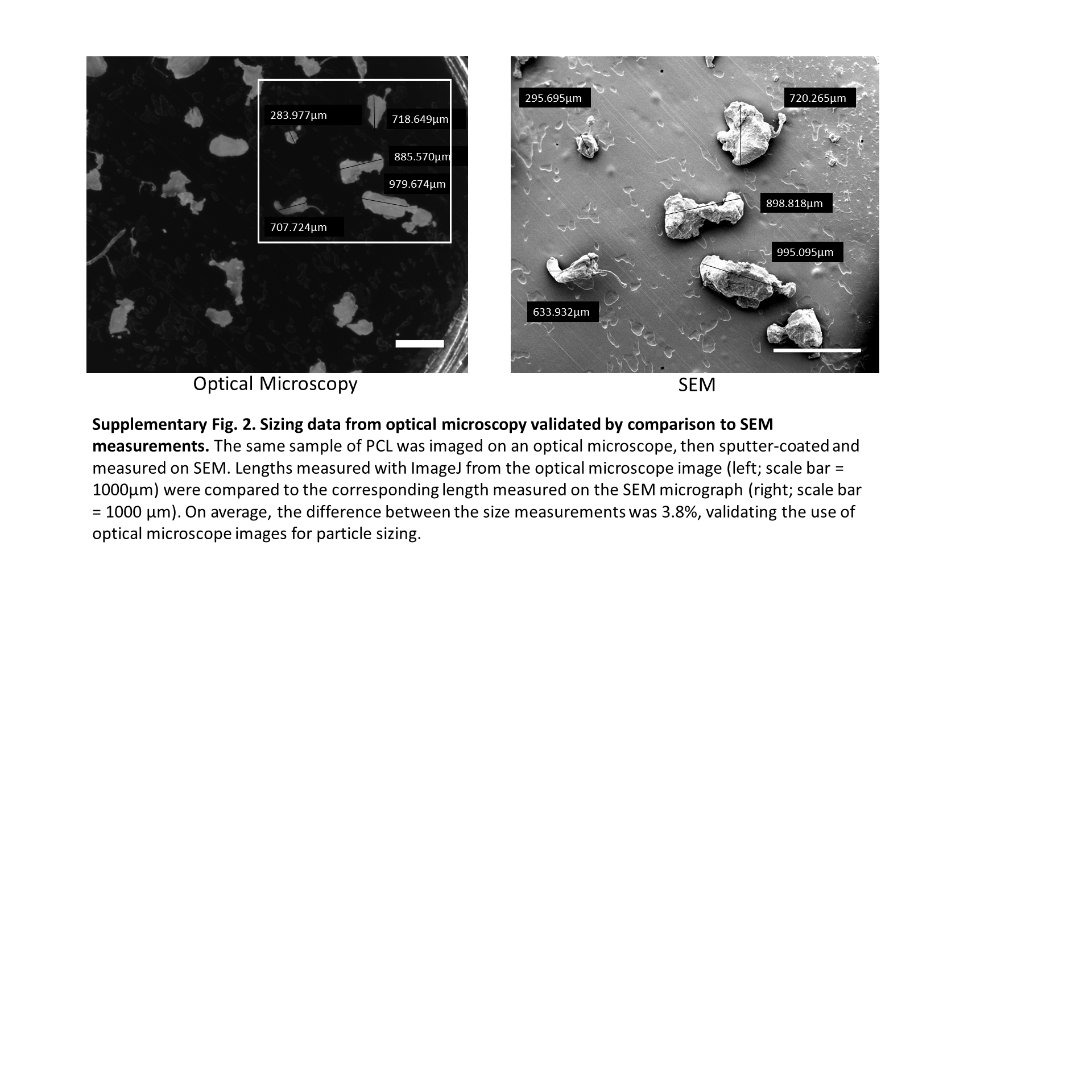

Supplement: S2 Fig — The same sample of PCL was imaged on an optical microscope, then sputter-coated and measured on SEM. Lengths measured with ImageJ from the optical microscope image (left; scale bar = 1000μm) were compared to the corresponding length measured on the SEM micrograph (right; scale bar = 1000 μm). On average, the difference between the size measurements was 3.8%, validating the use of optical microscope images for particle sizing. (TIF) [file pone.0147399.s004.tif]

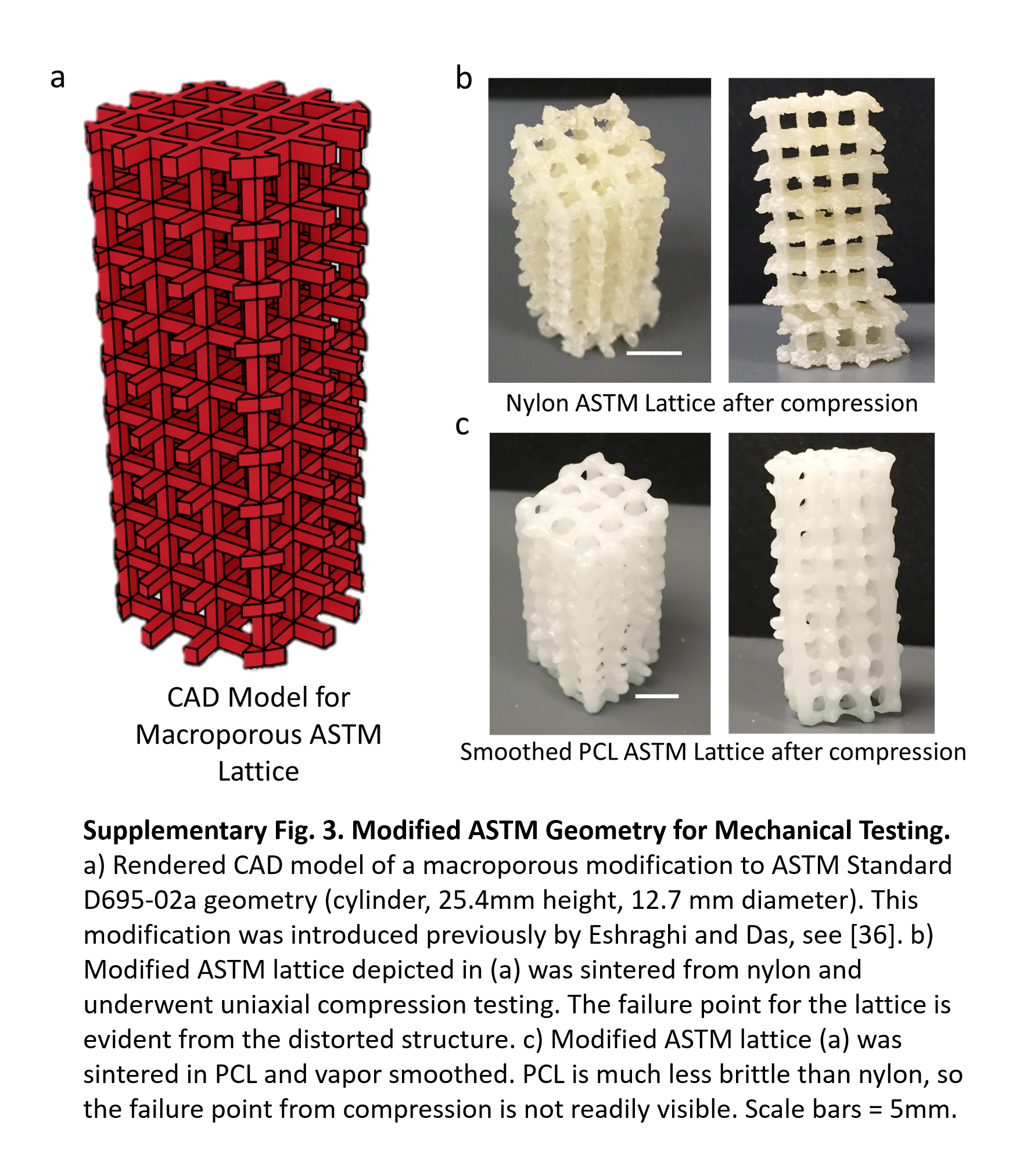

Supplement: S3 Fig — a) Rendered CAD model of a macroporous modification to ASTM Standard D695-02a geometry (cylinder, 25.4mm height, 12.7 mm diameter). This modification was introduced previously by Eshraghi and Das, see [36]. b) Modified ASTM lattice depicted in (a) was sintered from nylon and underwent uniaxial compression testing. The failure point for the lattice is evident from the distorted structure. c) Modified ASTM lattice (a) was sintered in PCL and vapor smoothed. PCL is much less brittle than nylon, so the failure point from compression is not readily visible. Scale bars = 5mm. (TIF) [file pone.0147399.s005.tif]
